# Diversity and Distribution of a Novel Genus of Hyperthermophilic *Aquificae* Viruses Encoding a Proof-Reading Family-A DNA Polymerase

**DOI:** 10.3389/fmicb.2020.583361

**Published:** 2020-11-12

**Authors:** Marike Palmer, Brian P. Hedlund, Simon Roux, Philippos K. Tsourkas, Ryan K. Doss, Casey Stamereilers, Astha Mehta, Jeremy A. Dodsworth, Michael Lodes, Scott Monsma, Tijana Glavina del Rio, Thomas W. Schoenfeld, Emiley A. Eloe-Fadrosh, David A. Mead

**Affiliations:** ^1^School of Life Sciences, University of Nevada, Las Vegas, Las Vegas, NV, United States; ^2^Nevada Institute of Personalized Medicine, University of Nevada, Las Vegas, Las Vegas, NV, United States; ^3^Department of Energy Joint Genome Institute, Berkeley, CA, United States; ^4^Department of Biology, California State University, San Bernardino, CA, United States; ^5^Lucigen Corporation, Middleton, WI, United States; ^6^Tamarack Bioscience, Inc., Beverly, MA, United States; ^7^Varigen Biosciences Corporation, Madison, WI, United States

**Keywords:** viral metagenomics, bacteriophage, uncultivated virus genome, UViG, geothermal spring, DNA polymerase

## Abstract

Despite the high abundance of *Aquificae* in many geothermal systems, these bacteria are difficult to culture and no viruses infecting members of this phylum have been isolated. Here, we describe the complete, circular dsDNA Uncultivated Virus Genome (UViG) of *Thermocrinis* Octopus Spring virus (TOSV), derived from metagenomic data, along with eight related UViGs representing three additional viral species. Despite low overall similarity among viruses from different hot springs, the genomes shared a high degree of synteny, and encoded numerous genes for nucleotide metabolism, including a PolA-type DNA polymerase polyprotein with likely accessory functions, a DNA Pol III sliding clamp, a thymidylate kinase, a DNA gyrase, a helicase, and a DNA methylase. Also present were conserved genes predicted to code for phage capsid, large and small subunits of terminase, portal protein, holin, and lytic transglycosylase, all consistent with a distant relatedness to cultivated *Caudovirales*. These viruses are predicted to infect *Aquificae*, as multiple CRISPR spacers matching the viral genomes were identified within the genomes and metagenomic contigs from these bacteria. Based on the predicted atypical bi-directional replication strategy, low sequence similarity to known viral genomes, and unique position in gene-sharing networks, we propose a new putative genus, “*Pyrovirus*,” in the order *Caudovirales*.

## Introduction

Viruses are the most abundant biological entities on Earth and are important drivers of genetic exchange, secondary production, and host metabolism on both local and global scales (Fuhrman, 1999; Suttle, 2007; Rohwer and Thurber, 2009; Breitbart et al., 2018). They also possess a high density of nucleic acid synthesis and modifying enzymes that are important sources of enzymes for the biotechnology sector. Despite their importance, cultivation of viruses in the laboratory is limited, partly by challenges associated with cultivating their hosts. This problem is particularly true for viruses of thermophiles and hyperthermophiles because many hosts remain uncultured (Hedlund et al., 2015; Lloyd et al., 2018). Also, most thermophiles do not readily form lawns on solid media, which are typically exploited to screen for viruses. Although direct observation of filtrates from geothermal springs and enrichments has revealed a high diversity of virus morphotypes (Rice et al., 2001; Rachel et al., 2002), few thermophilic viruses have been studied in enrichment cultures and even fewer have been isolated in culture with their host. Currently, the NCBI Viral Genomes database lists 59 thermophilic archaeal viruses out of 95 total genomes, representing ten families (accessed 2/3/20)^1^; however, 49 of these infect members of the thermoacidophilic family *Sulfolobaceae*, leaving other archaeal thermophiles vastly under-explored. Similarly, only 15 of the 2,500 bacteriophage genomes represent thermophilic or hyperthermophilic viruses, representing only three virus families (accessed 2/3/20)^2^. Strikingly, although members of the phylum *Aquificae* (syn. *Aquificota*) predominate in many terrestrial and marine high-temperature ecosystems (Reysenbach et al., 2005; Spear et al., 2005; Takacs-Vesbach et al., 2013), to date, no cultivated viruses infecting *Aquificae* have been described.

Microbial ecologists have increasingly turned to cultivation-independent approaches to probe microbial diversity in nature. Although the low nucleic acid content and lack of universal conserved marker genes slowed the development of viral metagenomics, this field is now in full swing (Paez-Espino et al., 2016; Emerson et al., 2018; Koonin and Dolja, 2018). One of the early viral metagenomic investigations focused on Octopus Spring and other circumneutral pH springs in Yellowstone National Park (Schoenfeld et al., 2008), revealing 59 putative DNA polymerase (*pol*) genes, which were subsequently screened for heterologous activity in *Escherichia coli* (Moser et al., 2012). The most thermophilic of these enzymes, 3173 PolA, also demonstrated high-fidelity, thermostable reverse-transcriptase (RT) activity and strand-displacement activity and was subsequently marketed by Lucigen Corporation as a single-enzyme RT-PCR system called PyroPhage and RapidDxFire. That enzyme was further improved by molecular evolution and fusion of a high-performance chimeric variant of 3173 PolA with the 5′ to 3′ exonuclease domain of *Taq* polymerase to improve probe-based detection chemistries and enable highly sensitive detection of RNA (Heller et al., 2019).

A study of the diversity and evolution of 3173 PolA and related polymerases revealed clues about its complex evolutionary history (Schoenfeld et al., 2013). In addition to their discovery in viral metagenomes from hot springs, 3173 *polA*-like genes were also detected in two of the three families of *Aquificae*, where they have orthologously replaced host DNA *polA* genes, and phylogenetically diverse, non-thermophilic bacteria, where they appear to be transient alternative *polA* genes, presumably due to recombination following non-productive infections. Amazingly, 3173 *polA*-like genes are also known to encode thermophilic, nuclear-encoded, apicoplast-targeted polymerases in eukaryotic parasites in the *Apicomplexa* (e.g., *Plasmodium*, *Babesia*, and *Toxoplasma*) (Seow et al., 2005). The origin of these genes likely involved fixation of a progenitor sequence into the nuclear genome following endosymbiosis of a red alga (proto-apicoplast) containing a bacterial symbiont carrying a viral *polA* gene (Schoenfeld et al., 2013).

Recently, an Uncultivated Virus Genome (UViG) containing the 3173 *polA* gene was described from metagenomic data (Mead et al., 2017) with preliminary comparisons to a limited number of related genomes, although the sequence data were not published. This study comprehensively interrogated new and existing metagenomes from terrestrial geothermal springs with the goal of obtaining additional UViGs related to the previously identified viral genome and to address several outstanding questions regarding this group of viruses: (i) what is their genomic diversity and what features are conserved and variable?, (ii) what is their environmental distribution?, (iii) what are their cellular hosts, and (iv) how are they related to other viruses? We uncovered nearly complete UViGs from several Yellowstone geothermal springs and Great Boiling Spring (GBS), Nevada that range from 37,256 to 41,208 bp and encode 48 to 53 open reading frames (ORFs). The presence of fragments of these viral genomes in CRISPR arrays encoded by *Thermocrinis ruber* OC1/4^T^, *Thermocrinis jamiesonii* GBS1^T^, *Hydrogenobaculum* sp. 3684, and *Sulfurihydrogenibium yellowstonense* SS-5^T^ genomes, along with similarity between many viral genes and *Aquificaceae* genes, supports the previous hypothesis (Schoenfeld et al., 2013; Mead et al., 2017) that *Thermocrinis* and probably other *Aquificae* are putative hosts of these viruses. The high abundance of these viruses and their hosts suggests they may play an important role in chemolithotrophic productivity in geothermal springs globally, in addition to their role in evolution as a vector for horizontal gene transfer.

## Materials and Methods

### Isolation of Uncultured Viral Particles From Octopus Hot Spring and Great Boiling Spring

Virus particles were isolated from Octopus Hot Spring in Yellowstone National Park (Permit # YELL-2007-SCI-5240), Wyoming (N 44.5342, W 110.79812) in 2007 and from Great Boiling Spring (GBS), Nevada, (N 44.6614, W 119.36622) in December 2008. Temperature at the time and location of sampling was 87°C at the outflow channel of Octopus Spring and 80–83°C in the source pool of Great Boiling Spring.

For Octopus Spring samples, thermal water (between 200 and 630 L) was filtered using a 100 kDa molecular weight cut-off (mwco) tangential flow filter (A/G Technology, Amersham Biosciences, GE Healthcare) and viruses and cells were concentrated to about 2 L. The resulting concentrates were filtered through a 0.2 μm tangential flow filter to remove microbial cells. The viral fractions were further concentrated to about 100 mL using a 100 kDa tangential flow filter and 40 mL of viruses were further concentrated to 400 μL and transferred to SM buffer (0.1 M NaCl, 8 mM MgSO_4_, 50 mM Tris HCl, pH 7.5) by filtration in a 30 kDa mwco spin filter (Centricon, Millipore).

For the GBS viral sample, tangential-flow filtration with a 30 kDa molecular weight cutoff Millipore Prep/Scale TFF-6 filter (catalog # CDUF006TT) was used to concentrate ∼500 L of GBS water to ∼2 L. Filtration was done in December 2008 with water from the GBS “A” site (Cole et al., 2013) with a temperature of 80–83°C and pH of 7.15-7.2. The concentrated sample was stored on ice and transported to the laboratory, where it was pelleted by centrifugation at 4°C for 10 min at 10,000 × *g*. The supernatant was then further concentrated as above, and the cell pellet was stored at −80°C for DNA extraction.

### Isolation of Viral and Planktonic Cell DNA

*Serratia marcescens* endonuclease (Sigma, 10 U) was added to both viral preparations described above to remove non-encapsidated (non-viral) DNA. The reactions were incubated at 23°C for 2 h. EDTA (20 mM), sodium dodecyl sulfate (SDS) (0.5%), and Proteinase K (100 U) were added and the reactions were incubated at 56°C. Subsequently, sodium chloride (0.7 M) and cetyltrimethylammonium bromide (CTAB) (1%) were added. The DNA was then extracted with chloroform, precipitated with isopropanol and washed with 70% ethanol. Yields of DNA ranged from 20 to 200 ng.

For preparation of cellular DNA from GBS, high molecular weight DNA was extracted from the pelleted cells essentially using the JGI bacterial DNA isolation CTAB protocol^3^. Briefly, this involved cell lysis with lysozyme (2.6 mg/mL), proteinase K (0.1 mg/mL), and SDS (0.5%), followed by purification of DNA by incubation with CTAB (1%) and sodium chloride (0.5 M), organic extraction, alcohol precipitation, treatment with RNase A (0.1 mg/mL), and an additional alcohol precipitation step.

### Whole-Genome Amplification of Viral Metagenomic DNA

For the viral library that contained sequences of OS3173, a linker-based amplification method was used as described (Schoenfeld et al., 2008). For subsequent viral preparation isolated viral metagenomic DNA was amplified with an Illustra GenomiPhi V2 DNA amplification kit (GE Healthcare, Piscataway, NJ, United States) following the manufacturer’s protocol. Briefly, 9 μL sample buffer and 1 μL sample DNA were mixed and incubated at 95°C for 3 min and then placed on ice. Nine microliter reaction buffer and 1 μL enzyme were then mixed and combined with the 10 μL sample and incubated for 2 h at 30°C and 10 min at 65°C. The amplified DNA was then precipitated with 0.2 M NaCl and 70% ethyl alcohol and resuspended in 40 μL water. The amplified DNA was debranched by adding 10 μL of 5X S1 nuclease buffer and 2 μL S1 nuclease (200 U; Thermo Fisher Scientific Inc., Waltham, MA, United States), mixed and incubated at 25°C for 30 min and then 70°C for 10 min. The sample was reprecipitated twice with 0.2 M NaCl and 70% ethyl alcohol and resuspended in 20 μL water. Several amplification reactions were prepared and used for DNA sequence analysis and to construct a large insert library in order to capture regions of the viral replisome.

### Metagenomic Sequencing and Assembly

The amplified Octopus Spring viral metagenomic DNA was sequenced using Roche 454 GS FLX Titanium chemistry at the Broad Institute (229,553 reads averaging 375 nucleotides each; 86,161,605 bases in total). The full read set was assembled *de novo* with CLC Genomics Workbench 8.0, using word size of 20 and bubble size of 375. A total of 5,143 contigs of length >500 were assembled with N50 = 1,818 bp, average length of 1,586 bp, maximum contig length of 35,614 bp, and total assembly length of 8,156,404 bp. Of the 229,553 original reads, 66% (152,673 reads) were incorporated into contig assemblies >500 bp.

**FIGURE 1:**
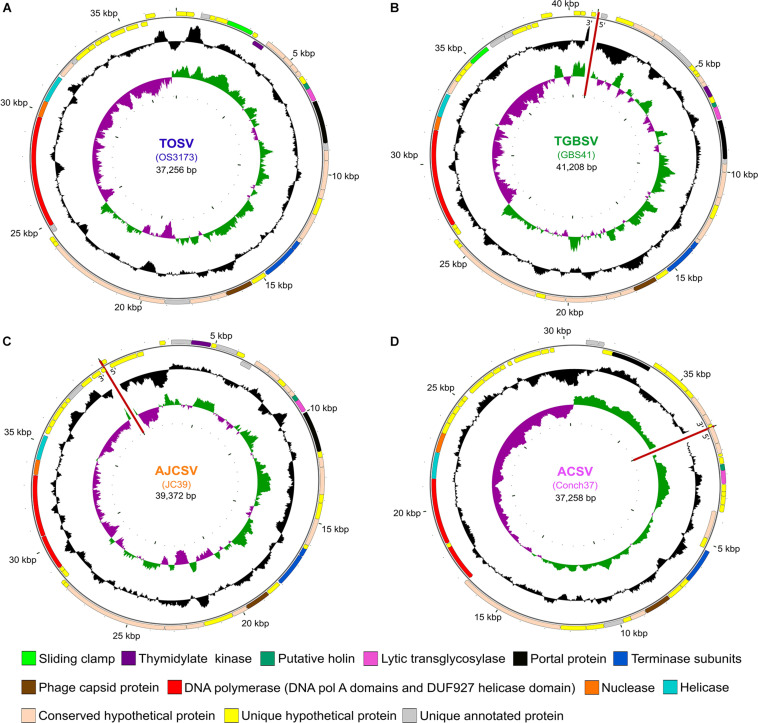
Map of four representative UViGs. The uncultivated viral genomes were recovered from metagenomes from **(A)** Octopus Spring (TOSV); **(B)** Great Boiling Spring (TGBSV); **(C)** Joseph’s Coat Spring (AJCSV); and **(D)** Conch Spring (ACSV). Outer circles show ORFs and selected annotation features, with arrows in the putative direction of transcription. Middle circles show the GC content and the inner circles show the GC skew. The sequences of GBS41, JC39, and Conch37 could not be circularized as indicated with red lines. Maps have been rotated to reflect the orientation of OS3173. TOSV, TGBSV, AJCSV, and ACSV are represented by OS3173, GBS41, JC39, and Conch37, respectively.

Of the reads, 56.6% (86,379 reads) mapped to the largest contig at a stringency of 90%, which eventually was closed as Octopus Spring OS3173 virus, resulting in an average coverage of 913-fold. The OS3173 consensus viral sequence was finished by an iterative process of extending the ends of this viral scaffold with partially mapped reads until the extended consensus ends were found to overlap. This resulted in a 37,256 bp circular genome. A total of 99,924 reads were mapped to the finished genome (also at 90% stringency), and reads were found to map continuously across the joined overlap, consistent with a circular topology. Reads that did not map at 90% stringency were saved and remapped at relaxed stringency (80% identity over 80% length). These relaxed stringency reads were found to contain structural variants. The origin of the reported viral sequence was arbitrarily set to the beginning of the first ORF clockwise of the negative to positive GC skew transition (Figure 1). Viral contigs with lower coverage from the virus-enriched metagenome were obtained by reassembling the same reads using SPAdes v. 3.13.1 (Bankevich et al., 2012) with default parameters, except for the option “–only-assembler.”

Both cellular and amplified viral metagenomes from GBS were sequenced at the DOE Joint Genome Institute using Roche 454 GS FLX Titanium chemistry. Double-stranded genomic DNA samples were fragmented via sonication to fragments ranging between approximately 400 and 800 bp. These fragments were end-polished and ligated to Y-shape adaptors during 454 Rapid Library Construction. Clonal amplification of the library fragments was then performed in bulk through hybridization of the fragments to microparticle beads and subsequent emulsion-based PCR. Beads containing amplified DNA fragments were loaded into wells of a Pico Titer Plate (PTP) so that each well contained a single bead, followed by sequentially flowing sequencing reagents over the PTP. For the water-borne cell metagenome, a total of 355,082 reads were obtained ranging in length from 56 to 2,049 nucleotides, with an average read length of 554 nucleotides, producing 196,771,207 bases in total. During preprocessing through the DOE-JGI Metagenome Annotation Pipeline [MAP; (Huntemann et al., 2016)]^4^, 454 reads shorter than 150 bp and longer than 1,000 bp were removed. The remaining reads were assembled with SPAdes v 3.6.1 (Bankevich et al., 2012), to a total of 315,164 contigs or sequences resulting in a total assembled size of 131,296,876 bases. Gene calling on the assembled sequences were done through the DOE-JGI MAP (Huntemann et al., 2016). Through this pipeline, CRISPR array prediction was also done and a total of 508 CRISPR arrays were found to be present in the GBS cell metagenome. After binning with the DOE-JGI binning pipeline, a single *T. jamiesonii* MAG was recovered. For the amplified viral metagenome or GBS virus-enriched metagenome, a total of 787,720 reads were sequenced ranging between 53 and 1,200 nucleotides for a total read library size of 392,631,172 bases. Read processing and assembly was also performed through the DOE-JGI MAP, in the same manner as the cellular metagenome. The virus-enriched metagenome had a total assembled size of 27,375,388 bases, which was divided over 55,185 contigs. In contrast to the cellular metagenome, only 137 RNA genes were predicted for this metagenome, supporting a low level of cellular contamination, and 74,087 protein-coding genes were predicted. A total of 60 CRISPR arrays were predicted.

### Functional Annotation

Open reading frames in OS3173 were identified by the GeneMarkS heuristic algorithm (Besemer et al., 2001). Open reading frames identified by GeneMarkS were submitted to NCBI BLASTP (Altschul et al., 1990) using default settings for comparison with proteins in the public database. The ORFs for all other UViGs were retained as identified through the DOE-JGI MAP (Huntemann et al., 2016).

Putative protein functions for four representative UViGs were inferred from searches against the NCBI non-redundant (nr) protein database with BLASTP using default settings^5^, NCBI Conserved Domain Database (CDD)^6^ with CD-Search, UniProtKB with HMMer^7^, and CDD, Protein Data Bank (PDB), SCOPe 70 and Pfam with HHPred^8^. An *E*-value cutoff of 1e^–10^ was used for all tools. For each tool, the result with the lowest *E*-value that was not a “hypothetical protein” was chosen as the putative function predicted by that tool (Stamereilers et al., 2018). In some instances, putative function was assigned by synteny and gene length (e.g., small subunit of the terminase, holin).

In order to compile a composite annotation for all four of the UViGs used as representatives of the four PolA species (i.e., “*Pyrovirus*”), all manual annotations were combined with functional annotations determined via the DOE-JGI MAP. Bidirectional BLASTP (Altschul et al., 1990) analyses were performed between all four viral representatives with default settings. Genes that were bidirectional best hits were considered homologous and robust annotations (separately identified as having the same function in at least two of the four UViGs) were transferred to all homologs. Where homologous genes had no functional annotation, or contradicting annotations between the reference sequences, the respective genes were denoted as encoding conserved hypothetical proteins.

### Single-Gene Trees

In order to place the viral sequences identified to be close relatives of OS3173 into phylogenetic context, two single-gene phylogenetic analyses were conducted on the protein sequences of firstly, the PolA from all viral scaffolds, together with the 3173 PolA-like sequences from Schoenfeld et al. (2013), and secondly, the large subunit sequence of terminase. For the PolA phylogeny, the 3173 PolA-like sequences of *Thermocrinis* species were used for outgroup purposes based on previous studies (Schoenfeld et al., 2013). In contrast, the terminase large subunit phylogeny was unrooted, and reference sequences of Chelikani et al. (2014), were used to infer the potential DNA packaging strategy of these viruses (Chelikani et al., 2014). Due to the variability present in these viral genes, the protein sequences were aligned based on structurally homologous protein domains with DASH (Rozewicki et al., 2019) in MAFFT v. 7 (Katoh et al., 2019)^9^, with default settings. The appropriate protein model of evolution was determined for the respective alignments with ProtTest 3.4 (Darriba et al., 2011) and maximum likelihood analyses were conducted with RAxML v. 8.20 (Stamatakis, 2014). Branch support for the phylogenies was inferred from 1,000 bootstrap pseudoreplicates.

### Prediction of Protein Domains

For the prediction of protein domains from the 3173 PolA-like sequences, a search of domain profiles based on hidden Markov Models was conducted through the EMBL-EBI hmmsearch tool^10^ against the pfam database (El-Gebali et al., 2019). Protein family domains were predicted for all 3173 PolA-like protein sequences used in this study to determine whether the DUF 927 helicase and DNA pol A exo domains are fused to the pol A domain of the 3173 PolA-like proteins to form a polyprotein. Transmembrane domains for putative holins present in the four representative genomes from the proposed genus “*Pyrovirus*” were predicted through the TMHMM server^11^.

### Genome Maps

Genome maps for the four reference sequences were constructed with CGView (Grant and Stothard, 2008)^12^. The GC content and skew for each genome was calculated with a step size of 1 bp using a sliding window of 500 bp. Protein-coding sequences were colored based on the homology inferences from the synteny analyses and the composite annotations for each genome. Breaks in the UViG sequences that were not circularized, i.e., GBS41, JC39, and Conch37, were indicated with red lines in all three tracks of the genome maps. The genome maps were rotated to align with that of OS3173 for easier visualization.

### Relative Abundance of Viral Contigs in Viromes

From the metagenomes analyzed, viral genomes were predicted with VirSorter v. 1.0.5 (Roux et al., 2015), Earth’s Virome pipeline (Paez-Espino et al., 2016), and Inovirus detector pipeline v. 1.0 (Roux et al., 2019b)^13^. From the respective viral-enriched metagenomes, 372 contigs were obtained with 42 contigs ≥10,000 bp. Dereplication was done with an Average Nucleotide Identity (ANI) of 95% over an alignment fraction of 85% to obtain 320 non-redundant contigs. Contig coverage was estimated by mapping reads from individual metagenomes to the 320 non-redundant viral contigs using BBMap v. 38.67^14^. Only reads that mapped at ≥95% nucleotide identity were considered and contig coverage was set at 0 if less than 70% of the contig’s length was covered by metagenomic reads, or as the average read depth per position otherwise, as typical for UViG analysis (Roux et al., 2019a).

### Viral Classification

All contigs ≥10,000 bp obtained from the virus-enriched metagenomes, together with the four representative UViGs, were used as input with the viral reference sequence database (RefSeq v94), to automatically delineate genus-level groups based on shared gene content in vContact2 using default parameters (Jang et al., 2019). The resulting gene-sharing network was viewed and edited in Cytoscape 3.7.2^15^, using a prefuse force directed layout.

### Proteomic Tree and Synteny Analyses

In order to confirm the relationships among the seven UViGs, a proteomic tree was constructed with ViPtree (Nishimura et al., 2017)^16^. This Neighbor-Joining (NJ) tree is constructed by computing genome-wide tBLASTx similarity scores (McGinnis and Madden, 2004) among all submitted and all reference viral sequences. From these similarity scores a distance matrix was obtained and used for constructing a BIONJ tree. Based on previous results, the nucleic acid type was specified as dsDNA, with prokaryotes indicated as the potential hosts. Gene predictions as performed above were used for the UViGs. This process was repeated for the 10 UViGs with the highest coverage in the two virus-enriched metagenomes (i.e., from Octopus Spring and Great Boiling Spring). For depicting synteny, the genome alignments based on tBLASTx analyses, as inferred with ViPtree, was used.

### Host Identification for Abundant Viruses in Great Boiling Spring and Octopus Spring

The ten viruses with the highest coverage in Great Boiling Spring and Octopus Spring, respectively, were identified from the viral metagenomes. A two-pronged approach was employed to identify potential hosts for these viruses. The first approach consisted of identifying potential prophages in bacterial and archaeal genomes, while the second approach consisted of identifying CRISPR spacers in host genomes matching the viral sequences.

For the identification of potential prophages matching the viral sequences, BLASTn analyses were conducted with the 10 viruses with the highest coverage in each spring to the DOE JGI/IMG isolate genome database (Chen et al., 2019), as well as the NCBI Whole Genome Shotgun (WGS) and RefSeq Genomic (refseq_genomic) databases.

For the second approach, CRISPR clusters were used from all metagenomes, single-amplified genomes (SAGs) and isolate genomes, available on IMG for Octopus Spring and Great Boiling Spring. All CRISPR spacer regions available on IMG for these genomes were used for further analysis. Those SAGs and isolate genomes that did not have CRISPR prediction results available on IMG were analyzed with CRISPRCasFinder (Couvin et al., 2018)^17^. All predicted spacer regions were then compared to the ten most covered virus sequences in each spring using BLASTn [BLAST v.2.2.31; (Altschul et al., 1990)] with custom settings (-word_size 7 -gapopen 10 -gapextend 2 -penalty -1 -outfmt 6 -dust no). For the spacer comparisons from the metagenomes, only spacer regions with matches over 100% of the length of the spacer were considered to limit the vast number of sequences interrogated, while matches over 80% of the length of the spacers were considered for SAGs and isolate genomes. Resulting BLAST hits were then further limited to those with a percentage identity of ≥80% and an Expect(*e*)-value of ≤0.00001.

For the CRISPR spacer detection of the four representative UViGs to *Hydrogenobaculum* sp. 3684 (Romano et al., 2013), *S. yellowstonense* SS-5^T^ (Reysenbach et al., 2009), *T. ruber* OC1/4^T^, and *T. jamiesonii* GBS1^T^, these microbial isolate genomes were subjected to CRISPR array prediction with CRISPRCasFinder. The resulting CRISPR arrays with a confidence level of three or above were further analyzed. All predicted spacer sequences were subjected to BLASTn analyses against OS3173, GBS41, JC39, and Conch37 as described above.

### Recruitment Plots

To visualize the level of variability within the viral populations and coverage across the UViGs for Octopus Spring and Great Boiling Spring, raw sequence reads were recruited to the UViGs of OS3173 and GBS41. The UViGs were used to construct BLAST databases using makeblastdb in BLAST v. 2.2.31. Following this, BLASTn analyses were conducted with each UViG database as reference and their respective metagenomic reads from which they were assembled, as query. Default settings for BLAST analyses were used apart from specifying tabular format for the data output (-outfmt 6), reporting a single HSP per subject sequence (-max_hsps 1), and keeping a single alignment per subject sequence (-max_target_seqs 1). The BLAST results were formatted with BlastTab.catsbj.pl^18^ limiting the identity of hits to report to 30%, and these data was then subjected to recruitment plot construction with enve.recplot2 in the Enveomics Collection (Rodriguez-R and Konstantinidis, 2016)^19^ in RStudio v. 3.6.1. To compare obtained recruitment plots to the genomic architecture of the UViGs, annotated UViGs were visualized with Geneious R7 (Biomatters) and edited in Inkscape v. 0.92.

### Sequence Accession Numbers

The individual sequence reads from the 2007 Octopus hot spring viral sample can be accessed at https://www.imicrobe.us/#/samples/345. The quality-filtered reads for the Octopus Spring metavirome and the Great Boiling Spring metavirome have been submitted to the National Center for Biotechnology Information (NCBI) Sequence Read Archive (SRA) with sequencing run accession numbers SRR12281643 and SRR12248450, respectively. The accession numbers for the twelve “*Pyrovirus*” contigs can be found in the DOE-JGI IMG/M (Chen et al., 2019) website^20^ under IMG Scaffold ID numbers found in Table 1. The four representative UViGs (for the species TOSV, TGBSV, AJCV, and ACSV) have also been submitted to the NCBI^21^ as third party assemblies under the nucleotide database, with accession numbers MK783188.1 (OS3173)">MK783188.1 (OS3173) and BK013345-7 (Conch37, JC39, and GBS41).

**TABLE 1:** Distribution of OS3173-like *polA* genes in metagenomic databases.

	Hot spring^*a*^	Temp. (°C)	pH	% AA ID^*b*^	Largest scaffolds (kbp)	Genbank or IMG accession
Yellowstone National Park	Octopus	85	8.0	82–90	37, 28	MK783188.1, JGI20132J14458_1000016
	Conch	85	8.8	66–91	37, 32	Ga0080008_153848, Ga0080008_158027
	Joseph’s Coat	74	2.5	25–37	39	Ga0080003_1000231
	Bath	85	8.0	70–94	1	2007311021
	Black Pool	73	8.0	56–89	8	Ga0111098_10004
	Calcite	75	7.8	39–49	32	YNPsite12_CeleraDRAF_ scf1119014592999
	Bechler	81	7.8	83–92	0.7	YNPsite13_CeleraDRAF_29640
United States Great Basin	Great Boiling	80	6.4	35–50	41	Ga0097684_1000009
	Sandy’s West	86.6	7.0	34–57	7	Ga0105155_1001723
	Little Hot Creek	82	6.8	33–50	2	Ga0105158_1016092

*^*a*^Metagenomes from YNP (Inskeep et al., 2013; Takacs-Vesbach et al., 2013; Inskeep and Jay, unpl.). ^*b*^Range of amino acid identities to the full-length OS3173 Pol based on tBLASTx.*

## Results and Discussion

### Dominant UViGs From Octopus Spring and Great Boiling Spring Encode an Unusual DNA Polymerase

**FIGURE 2:**
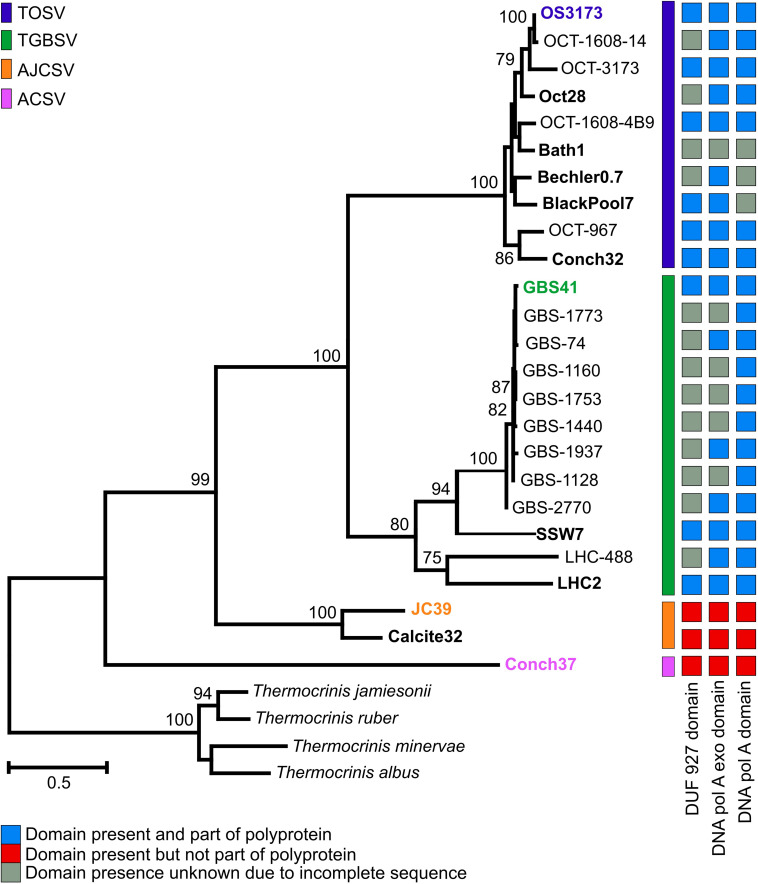
Phylogeny and structure of 3173 PolA-like proteins. Maximum-likelihood phylogeny of near full-length 3173 PolA-like proteins, with bootstrap values above 70% from 1,000 pseudoreplicates indicated. OCT, Oct or OS, Octopus Spring; Conch, Conch Spring; Bath, Bath Spring; Bechler, Bechler Spring; BlackPool, Black Pool; GBS, Great Boiling Spring; SSW, Sandy’s Spring West; LHC, Little Hot Creek; JC, Joseph’s Coat Spring; Calcite, Calcite Spring. The presence of helicase, exonuclease, and polymerase domains are indicated, where known, and whether these domain forms part of the polyprotein. The exonuclease and polymerase domains of AJCSV and ACSV are present and fused as a single ORF. The scale bar indicates the number of amino acid changes per site. Taxa indicated in bold represent contigs obtained from metagenomes, while the representative UViG of species group is colored in the corresponding color.

Viral particles were isolated from Octopus Spring in Yellowstone National Park and Great Boiling Spring (GBS) in the United States Great Basin by sequential tangential-flow filtration (Schoenfeld et al., 2008) and used for metagenomic sequencing (Mead et al., 2017). In parallel, the cell fraction from GBS was also used for metagenomic sequencing. Forty-three percent of the reads from the Octopus Spring virus-enriched metagenome assembled into a single contig herein called OS3173 [representative of the proposed species *Thermocrinis* Octopus Spring virus or TOSV, equivalent to the term OS3173 used previously (Mead et al., 2017)]. The OS3173 genome was 37,256 bp and encoded 48 predicted open reading frames (Figure 1A and Supplementary Figure S1A), as detailed below. Metagenomic sequence coverage was high (mean 913X) and uniform across the OS3173 genome above 95% nucleotide identity, and read depth was low at lower identity (Supplementary Figures S1B,C). Together, these data indicate that TOSV was likely the dominant virus present at the time and place of sampling. Among the 48 predicted genes was a full-length, PolA-type DNA polymerase polyprotein nearly identical to 3173 PolA (OCT-3173; Figure 2), a portion of which was previously discovered via Sanger sequencing of metagenomic clone libraries (Schoenfeld et al., 2008). The near-complete absence of any TOSV reads from a pink streamer microbial metagenome dominated by *Thermocrinis* from the outflow of Octopus Spring (Takacs-Vesbach et al., 2013) suggests viral activity is temporally or spatially variable in that environment, or that the virus has a lytic lifestyle that results in lysed cells that are rapidly cleared from the pink streamer community in the outflow channel.

**FIGURE 3:**
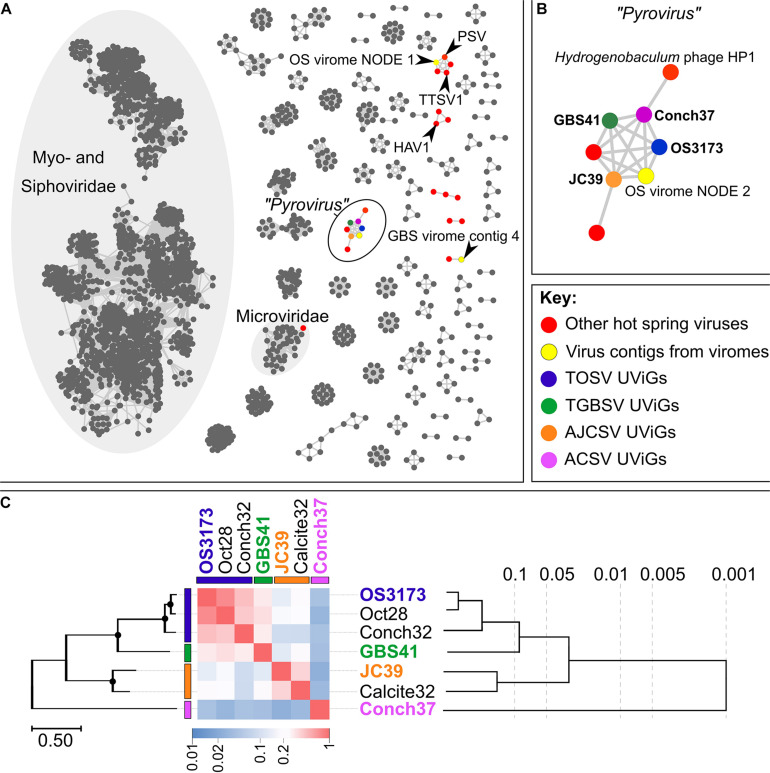
Relatedness inferred from gene-content between the OS3173-like UViGs. **(A)** Gene-sharing network inferred by vContact2 and visualized with Cytoscape 3.7.2. Nodes in the network represent cultivated or uncultivated viral genomes, while edges represent shared gene content between nodes. Viral contigs identified from hot spring microbial genomes are indicated in red, while contigs from either the Octopus Spring or Great Boiling Spring virus-enriched metagenomes are indicated in yellow (see Supplementary Figures S2, S3, S5, S6 and Supplementary File S1). The four representative UViGs are color-coded in their respective species colors. PSV, *Pyrobaculum* spherical virus; TTSV1, *Thermoproteus tenax* spherical virus 1; HAV1, Hyperthermophilic Archaeal virus 1. **(B)** Component of the gene-sharing network [circled in black on **(A)**] representing “*Pyrovirus*,” connecting the four representative UViGs together with two viruses peripheral to the network, *Hydrogenobaculum* phage HP1 and another uncultivated virus from a pink streamer microbial community metagenome from Octopus Spring, with each only sharing gene content with a single representative of the larger group. One additional viral contig of the Octopus Spring virus-enriched metagenome was connected to the genus-level group “*Pyrovirus*” (OS virome NODE 2). **(C)** Genomic relatedness among the seven related UViGs based on normalized tBLASTx scores across the genomes (heatmap) with the PolA phylogeny depicted on the left of the figure and a BioNJ phylogeny inferred from tBLASTx scores on the right. Bootstrap values above 95% on the PolA phylogeny are indicated with circles at nodes. The phylogeny based on normalized tBLASTx scores of these UViGs, and their placement within the dsDNA viral reference sequences database, is indicated in Supplementary Figure S6. OS3173, GBS41, JC39, and Conch37 represent TOSV, TGBSV, AJCSV, and ACSV, respectively.

Other viral contigs with lower coverage present in the Octopus Spring virus-enriched metagenome (Figure 3 and Supplementary Figures S2, S3) were similar to *Pyrobaculum* Spherical Virus (PSV) (Häring et al., 2004), a member of the *Globuloviridae*, which was previously described in Octopus Spring viral metagenomes (Schoenfeld et al., 2008; Mead et al., 2017), or distantly related to *Siphoviridae* viruses infecting mesophilic *Actinobacteria* or *Leptospira* (Supplementary Figure S3 and Supplementary File S1).

A similar viral contig encoding a 3173 PolA-like protein (Figure 2), GBS41, representative of the putatively named species *Thermocrinis* Great Boiling Spring virus (TGBSV), was obtained from the GBS cell metagenome. The GBS41 genome is 41,208 bp and encodes 53 putative open reading frames (Figure 1B and Supplementary Figure S4A). Metagenomic sequence coverage was low across the majority of the genome (mean 15.4X), yet it was highly variable in the intergenic regions on either end of the linear contig (Supplementary Figures S4B,C). Metagenomic reads were also recruited from the GBS virus-enriched metagenome at 50.4X coverage, where TGBSV had the highest coverage (Supplementary File S1), although the *de novo* assembly was not complete. In contrast to Octopus Spring, the high recruitment of viral reads from the GBS cellular metagenomes suggests active infection of *T. jamiesonii* in GBS during the time of sampling. This hot spring community is primarily planktonic and the long residence time of the GBS source pool (Costa et al., 2009) may allow for the capture of active viral infections, unlike the rapidly flowing streamer community analyzed in Octopus spring.

Other contigs from the GBS virus-enriched metagenome (Supplementary Figures S5, S6) were distantly related to viruses from halophilic *Euryarchaeota*, various *Sulfolobales* viruses, and PSV (Supplementary Figure S6). *Pyrobaculum* is relatively abundant in GBS (Costa et al., 2009; Cole et al., 2013); however, *Sulfolobales* are not known to occur at GBS, as no high-temperature, low-pH habitat is known to exist there. Due to the small size of these contigs and large genetic distance to characterized relatives, these relationships are highly uncertain.

The virus-enriched metagenomes from Octopus Spring and GBS are summarized in Supplementary File S1, including read recruitment, vContact 2.0 files, and CRISPR spacer matches of the 10 viral contigs with the highest coverage from these metagenomes.

### Recovery of OS3173-Like Genomes From Yellowstone and Great Basin Spring Metagenomes

To assess the distribution and diversity of OS3173-like viruses, the full-length 3173 *polA* gene of TOSV was used to recruit homologs *in silico* from public databases. In total, 12 unique contigs containing 3173 *polA*-like genes were obtained from whole-community and virus-enriched metagenomes from Yellowstone National Park and Great Basin hot springs (Table 1 and Figure 2 and Supplementary Figure S7). Thus, all UViGs from sources other than the Octopus Spring and Great Boiling Spring viromes were obtained from publicly available data. The Yellowstone springs from which OS3173-like UViGs were recovered, specifically Octopus Spring, Conch Spring, Joseph’s Coat Spring, and Calcite Spring, span several geothermal areas; the pH ranges from 2.5 in this specific Joseph’s Coat spring (JC2E) to 8.8 at Conch spring, originate from boiling or near-boiling spring discharge, and are known to host abundant populations of *Aquificae* (Reysenbach et al., 1994, 2000; Inskeep et al., 2010; Takacs-Vesbach et al., 2013) (Inskeep unpl.). In the Great Basin, Great Boiling Spring and Sandy’s Spring West are only ∼1 km apart (Costa et al., 2009), but Little Hot Creek is ∼380 km away, and each is separated from the Yellowstone springs by >1,200 km. These springs also share a circumneutral pH, near-boiling sources, and abundant *Aquificae* populations (Costa et al., 2009; Vick et al., 2010; Cole et al., 2013).

Phylogenetic analysis of the near-complete 3173 PolA-like proteins revealed four well-supported groups (representing putative novel species) that were mostly site-specific (Figure 2), except that one of two Pols from Conch Spring grouped with several from Octopus Spring in TOSV, whereas a distinct Conch Spring Pol split off at the most basal node in the phylogeny (*Aquificae* Conch Spring Virus, ACSV). Additionally, the Pols from the two springs, Joseph’s Coat Spring and Calcite Spring, grouped together in *Aquificae* Joseph’s Coat Spring Virus (AJCSV). The Pols from Great Basin springs were monophyletic and distinct from the Yellowstone Pols, forming TGBSV, following a pattern seen for several thermophilic bacteria and archaea (Miller-Coleman et al., 2012; Dodsworth et al., 2015; Zhou et al., 2020). All the full-length 3173 PolA-like proteins contained a 3′-5′ proofreading exonuclease and DNA polymerase (3′exo/pol) domain, as is typical of many bacterial PolAs. Several also contained putative helicase domains (DUF927), described later in detail; however, this domain was fused to form a putative polyprotein in TOSV and TGBSV, or alternatively present as a separate open reading frame in the three most divergent Pols, all from springs north of Yellowstone Lake (AJCSV and ACSV) (Figure 2). Each of the metagenomes contained only one of the Pol variants, except for the previously mentioned Conch Spring Pols.

**FIGURE 4:**
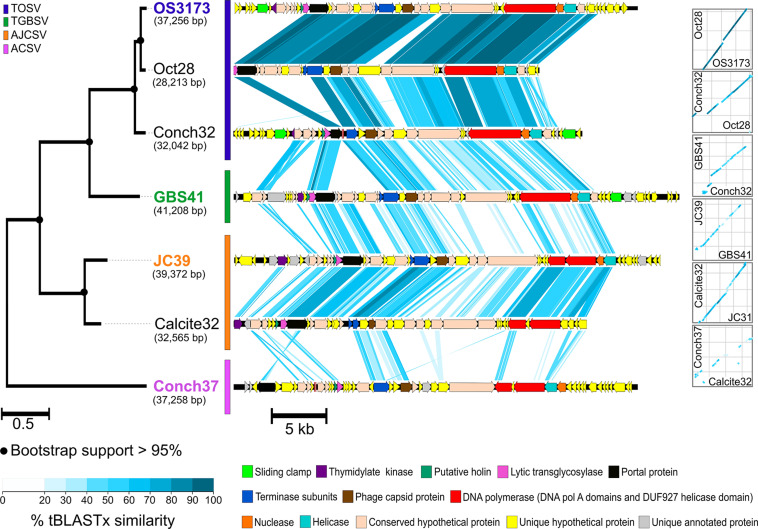
Synteny and amino acid identity across OS3173-like viral genomes. Synteny based on pairwise tBLASTx similarity across OS3173-like viral genomes, showing high overall synteny and few inversions, despite low amino acid identity between groups. The PolA phylogeny for the seven UViGs is indicated on the left of the figure. The representative taxa of each PolA-based species group are indicated in the species’ corresponding color. Bootstrap support above 95% is indicated at the nodes. On the right of the figure dot plots representing overall genomic synteny between the UViGs are indicated. OS3173, GBS41, JC39, and Conch37 represent TOSV, TGBSV, AJCSV, and ACSV, respectively.

Seven of the 12 contigs containing the genes encoding the 3173 PolA-like proteins were >20 kbp and were thus considered UViGs (Figure 4 and Table 2 and Supplementary Table S1). All seven UViGs were compared by tBLASTx to identify other regions of homology and assess genomic synteny (Figure 4). Within the groups previously identified by the Pol phylogeny, shared gene content and synteny were both high. Between the groups, shared gene content and synteny was considerably lower, reflecting low average amino acid identities (Figure 3C); however, some of the core genes were organized similarly even in the most distant genomes, including the polymerase/helicase, terminase subunits, and phage capsid protein, described in detail below.

**TABLE 2:** Summary of genomic features from four representative viral UViGs.

UViG	Source	Length	%GC	Number of genes	% Coding	Annotated proteins	% Annotated proteins
TOSV (OS3173)	Octopus Spring, WY	37,265	37.1%	49	95.1%	21	35%
TGBSV (GBS41)	Great Boiling Spring, NV	41,208	36.9%	53	94.5%	19	36%
AJCSV (JC39)	Joseph’s Coat Spring, WY	39,372	34.0%	51	96.5%	17	33%
ACSV (Conch37)	Conch Spring, WY	37,258	35.5%	50	94.8%	15	30%

For the classification of these seven UViGs, vContact2 was used to delineate genus-level groups for four representatives, one from each species group in the Pol phylogeny, consisting of TOSV (OS3173), TGBSV (GBS41), AJCSV (JC39), and ACSV (Conch37) (Figure 1 and Table 2). These four representatives were selected because they were considered complete or near-complete and thus allow more robust classification through gene-sharing networks (Jang et al., 2019). The four representative UViGs were connected as a single component of the gene-sharing network (Figure 3A), with representatives from all four groups forming a single putative genus (proposed “*Pyrovirus*”) (Figure 3B). One peripheral member of the “*Pyrovirus*” network (i.e., shared gene content detected only with ACSV) was *Hydrogenobaculum* phage HP1 (Figures 3A,B and Supplementary Figures S8, S9; Gudbergsdóttir et al., 2016), a 19,351 bp UViG recovered from a metagenome from Grensdalur, Iceland that was assigned to *Hydrogenobaculum* based on CRISPR spacer matches to genomes from cultivated *Hydrogenobaculum* strains. A second peripheral member of the “*Pyrovirus*” network (below the “*Pyrovirus*” group, shared gene content only detected to AJCSV; Figure 3B) was obtained from a microbial metagenome of a pink streamer community from Octopus Spring. The gene-sharing network also illuminated some other viral contigs from the Octopus Spring and GBS viromes, belonging to gene-sharing sub-networks with PSV and *Thermoproteus tenax* spherical virus 1 (TTSV) (Ahn et al., 2006), Hyperthermophilic archaeal virus 1 (HAV) (Garrett et al., 2010), and *Microviridae*, among other isolated clusters. No genomes belonging to the primary *Myoviridae* or *Siphoviridae* networks were present in the hot spring metagenomes, reflecting the unique gene content of hyperthermophilic viruses.

### Bi-Directional Genome Replication Strategy and Unique Genomic Features

The four representative genomes (OS3173, GBS41, JC39, and Conch37) ranged from 37,256 to 41,208 bp in length, ranged in GC content from 34.0 to 37.1%, and encoded 48 to 53 open reading frames, with coding fraction ranging from 94.5 to 96.5% (Table 2, annotations found in Supplementary Files S2, S3). The OS3173 contig assembled into a circular genome, whereas the other genomes could not be circularized (Table 3), possibly due to lower coverage or incomplete assembly owing to population heterogeneity. For now, it is uncertain whether the genomes represent circular genomes or linear genomes with terminal repeats.

**TABLE 3:** Minimum information about uncultivated virus genomes (MIUViG) for the four representative UViGs.

Metadata	TOSV (OS3173)	TGBSV (GBS41)	AJCSV (JC39)	ACSV (Conch37)
Source of UViG	Viral fraction metagenome (virome)	Metagenome (not viral targeted)	Metagenome (not viral targeted)	Metagenome (not viral targeted)
Sequencing approach	454 GS FLX Titanium	454 GS FLX Titanium	Illumina HiSeq 2000, 2500	Illumina HiSeq 2000, 2500
Assembly software	CLC Genomics 8.0 (word size = 20, bubble size = 375), SPAdes v3.13.1	SPAdes v 3.6.1	SPAdes v 3.10.0 (–meta –only-assembler -k 21, 33, 55, 77, 99, 127)	SPAdes v 3.10.0 (–meta –only-assembler -k 21, 33, 55, 77, 99, 127)
Viral identification software	VirSorter, Earth’s Virome pipeline, Inovirus detector pipeline	VirSorter, Earth’s Virome pipeline, Inovirus detector pipeline	VirSorter, Earth’s Virome pipeline, Inovirus detector pipeline	VirSorter, Earth’s Virome pipeline, Inovirus detector pipeline
Predicted genome type	dsDNA	dsDNA	dsDNA	dsDNA
Predicted genome structure	Non-segmented	Non-segmented	Non-segmented	Non-segmented
Detection type	Independent sequence (UViG)	Independent sequence (UViG)	Independent sequence (UViG)	Independent sequence (UViG)
Assembly quality	Finished	High-quality draft	High-quality draft	High-quality draft
Number of contigs	1	1	1	1

For all genomes, the transcriptional orientation of the ORFs is generally divided into a 23–26 kb set of contiguous genes on the same strand (clockwise in Figure 1), encoding 32–37 genes, and a smaller block on the other strand (counterclockwise in Figure 1), encoding 13–18 genes. As with most viral genomes, most genes are located in large blocks on the same strand. In each genome there are two to four instances of changes of strand involving one to two genes, except GBS41, which consists exclusively of two large gene blocks, one on each strand. In the Conch37 genome, there are two instances of a change of strand, each consisting of two genes. In each genome, a small (750–1,350 bp) intergenic region separated the sets of divergently transcribed genes, and this intergenic region also marked a strong divergence in GC skew. These features suggest bidirectional DNA replication beginning in the intergenic region around 36,429 bp of OS3173 and the corresponding regions of the other viral genomes. These intergenic regions also contained repetitive elements predicted to form stem-loop structures, consistent with secondary structures typical of origins of replication. Many bacterial genomes are replicated bidirectionally, and their genomes have a G > C bias in the leading strand of replication and a C > G bias in the lagging strand (Képès et al., 2012); however, dsDNA phage do not typically replicate bidirectionally (Weigel and Seitz, 2006), and in this regard we suggest these viral genomes replicate more like mini bacterial genomes than typical phage genomes. Examples of dsDNA viruses replicating bidirectionally include T7, lambda, and P4 phages, while T4 and P1 have bidirectional replication phases (Weigel and Seitz, 2006). However, cultivation of one of the viruses would be necessary to test this hypothesis.

The presence of polymerase-, nuclease/ recombinase-, and helicase-annotated genes in the smaller, counterclockwise set of genes in all four genomes suggests these genes might be transcribed earlier than the mainly structural genes in the larger, clockwise-facing block (Figure 1 and Supplementary File S2). However, some genes encoding proteins associated with nucleotide metabolism were located among the clockwise-facing genes, including a DNA Pol III beta subunit (sliding clamp) in OS3173; a thymidylate kinase in OS3173, GBS41, and JC39; and several genes that were found in only one of the four genomes, including site-specific DNA methylase (JC39), ribonucleotide reductase beta subunit (JC39), ATPase/kinase (JC39), and methyltransferases (Conch37). The location of these genes among the clockwise-facing part of the genomes and variability of these genes among the four UViGs suggest a variable and complex transcriptional/replication lifecycle for these viruses, or alternatively, that some nucleotide modification may be required during the lytic phase of infection.

Several genes encoding enzymes putatively involved in nucleic acid metabolism or DNA replication bear similarity to those in other viruses. ORF 3 of OS3173 encodes a 119-amino acid protein with some similarity to a *Sulfolobus* virus anti-CRISPR protein (Acr) (Athukoralage et al., 2020) that is highly conserved in diverse viruses and plasmids (Keller et al., 2007; Larson et al., 2007; Athukoralage et al., 2020). OS3173 and GBS41 both encode a putative sliding clamp beta subunit of DNA polymerase III, but they both lack an obvious clamp loader. Whether the viral replicase uses the host clamp loader or encodes an unrecognized clamp loader is unknown. Other viruses, including bacteriophage T4, encode sliding clamps, which have been shown to greatly increase processivity and the rate of replication (Trakselis et al., 2001). OS3173, GBS41, and JC39 each encode putative thymidylate kinases. Thymidylate kinases are encoded by a variety of viruses, including T4 and herpes simplex type 1 viruses. They are part of the nucleotide salvage pathway, typically have broad substrate activity, and are popular targets for antiviral drugs as they are often required for viability (Xie et al., 2019). ORF 5 in JC39 encodes a putative site-specific DNA methylase. Viral genome methylation is a common epigenetic defense against host restriction-modification systems. Two putative methyltransferases of unknown activity are encoded by ORF 41 and ORF 42 of Conch37.

The counterclockwise-oriented genes included three major replicase-associated proteins that were conserved in all four UViGs: an ATP-dependent helicase (ORF 38 in OS3173), a nuclease/recombinase (ORF 37 in OS3173), and a large polyprotein encoding a Pol A with functionally active polymerase activity (OS3173 Pol) (ORF 36 in OS3173). The helicase genes contain two P-loop-containing nucleoside triphosphate hydrolase domains related to the DEAD-like helicase superfamily, but the similarity to functionally characterized homologs is low. The Cas4-RecB-like nuclease (ORF 37 in OS3173) belongs to the PD-(D/E)XK nuclease superfamily, and may function as a single-stranded DNA-specific nuclease during replication and/or recombination, as these functions have been demonstrated for similar enzymes encoded by thermophilic archaeal viruses (Gardner et al., 2011; Guo et al., 2015).

In OS3173, ORF36 encodes the 1,606-amino acid polyprotein (OS3173 Pol), which was used to identify this group of viruses in the metagenomes (Figure 2). The amino-terminal region has conserved motifs that suggest primase and/or helicase function, including DUF927 (conserved domain with carboxy terminal P-loop NTPase) and COG5519 [Superfamily II helicases associated with DNA replication, recombination, and repair (Marchler-Bauer et al., 2010)]. Consensus Walker A and Walker B motifs suggest NTP binding and hydrolysis likely associated with helicase activity (Walker et al., 1982). As reported previously (Schoenfeld et al., 2013), the viral *polA* genes are similar to the single genomic *polA* of *Aquificaceae* and *Hydrogenothermaceae*, as well as genes found as additional *polA* copies in a variety of other bacterial genomes, and to the nuclear-encoded, apicoplast-targeted DNA polymerases of several *Apicomplexa* species, typified by the Pfprex protein of *Plasmodium falciparum*. That enzyme is also encoded as a polyprotein that is proteolytically processed to a polymerase that is optimally active at 75°C (Seow et al., 2005), much higher than would be encountered during the *Plasmodium* life cycle, but similar to the optimal growth temperature of *Thermocrinis* and the geothermal springs sampled in this study, implying lateral gene transfer (Schoenfeld et al., 2013). Understanding the biochemical functions of the rest of the ORF 36 domains could reveal new thermostable accessory proteins for DNA amplification.

Most of the clockwise-facing genes that were annotated suggest these UViGs are dsDNA tailed viruses belonging to the *Caudovirales*. Independent evidence that these viruses have dsDNA genomes comes from the initial study reporting the OS3173 PolA (Schoenfeld et al., 2008), because the viral DNA was amplified using a linker-dependent PCR method that is specific for dsDNA. Furthermore, OS3173 ORF 25, along with corresponding genes in the other UViGs, was annotated as a terminase large subunit, and ORF 24 was inferred to be a terminase small subunit, based on location immediately upstream of the terminase large subunit, open reading frame length (∼300–400 bp), and a similar isoelectric point as other terminases. The terminase small subunit protein allows recognition of the packaging site in the viral genome through specific binding of the DNA (Kala et al., 2014). Terminase large subunit phylogenies have previously been used to infer the viral DNA packaging mechanism (Chelikani et al., 2014; Merrill et al., 2016); however, the terminase large subunits from this group of viruses were distant from those of well-studied viruses, so the DNA packaging mechanism could not be inferred by this method (Supplementary Figure S10). Downstream of the putative terminase subunits in all genomes is a putative phage capsid protein at ORF 27 in OS3173. ORF 16 in OS3173 was annotated as a portal protein, which forms dodecameric rings that play critical roles in virion assembly, DNA packaging, and DNA injection in *Caudovirales* (Prevelige and Cortines, 2018). Additionally, GBS41 encodes a putative prohead protease (ORF 1), a WAIG tail domain protein (ORF 3), and a T7 tail fiber protein homolog (ORF 5), further supporting a relationship to *Caudovirales* and suggesting it encodes tail fibers typical of many *Caudovirales*. ORF 15 in OS3173 was annotated as a lytic transglycosylase (lysin) based on the presence of a lysozyme-like domain. ORF 14 in OS3173 was annotated as a holin based on the presence of three transmembrane domains, its small size (270 bp), and its location immediately upstream of ORF15. Also, the overlapping of open reading frames between ORFs 13, 14, and 15, suggests an anti-holin, holin, lysin operon, as found in numerous viruses. Together, these enzymes form the lysis cassette, which is common in *Caudovirales* bacteriophage, but for which there is limited knowledge in viruses of *Archaea* (Prangishvili, 2013; Saier and Reddy, 2015). No lysogeny-related genes [e.g., integrases, excisionases or Cro/CI genes (Lima-Mendez et al., 2011; Shao et al., 2017)] were identified from these UViGs, suggesting a purely lytic lifestyle. This was also supported by the fact that no prophage sequences could be identified from any microbial genomes. As most of the clockwise-facing genes appear to be involved in viral packaging and lysis, these genes are predicted to be transcribed later than the counterclockwise-facing genes, as the lysis cassette is typically the last to be transcribed (Labrie et al., 2004; Young, 2014).

Each of the UViGs encode numerous hypothetical proteins with no predicted function (∼70%; including hits to known hypothetical proteins as well as those with no homology to known proteins; Supplementary File S2), as is common in bacterial and archaeal viruses (Gardner et al., 2011; Gong et al., 2017; Zhang et al., 2018; Wang et al., 2019). Several of these were conserved among the genomes, but others were unique to each genome, or have diverged sufficiently that primary sequence conservation is difficult to discern. Many of the hypothetical proteins are related to genes found in different members of the *Aquificae* (Supplementary File S2), consistent with the previous hypothesis that *Thermocrinis* and possibly other *Aquificae* are the putative hosts for these viruses.

### Putative Hosts Belong to the *Aquificae*

**FIGURE 5:**
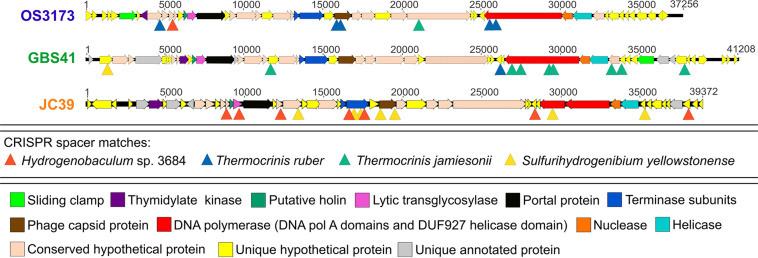
CRISPR spacers from *Aquificae* genomes matching the “*Pyrovirus*” genomes. Linearized maps of OS3173, GBS41, and JC39 genomes with sites matching *Thermocrinis ruber* OC1/4^T^, *Thermocrinis jamiesonii* GBS1^T^, *Hydrogenobaculum* sp. 3684 CRISPR and *Sulfurihydrogenibium yellowstonense* SS-5^T^ spacer sequences are denoted by triangles. OS3173, GBS41, and JC39 represent TOSV, TGBSV, and AJCSV, respectively. Detailed information on the CRISPR spacers can be found in Supplementary Figure S11.

Arrays of Clustered Regularly Interspaced Palindromic Repeats (CRISPRs) and related Cas (CRISPR associated) genes found in many bacterial and archaeal genomes (Grissa et al., 2007) provide a means to infer virus-host relationships (Heidelberg et al., 2009; Snyder et al., 2010; Anderson et al., 2011; Gudbergsdóttir et al., 2016; Roux et al., 2019b), as the CRISPR spacers provide a record of foreign nucleic acids that have been recorded by the CRISPR-Cas system. To determine the potential host range of these UViGs, genomes derived from isolates of *Hydrogenobaculum* sp. 3684, *S. yellowstonense* SS-5^T^, *T. ruber* OC1/4^T^, and *T. jamiesonii* GBS1^T^ were screened for CRISPR arrays to identify spacers matching the UViGs. Of the four bacterial genomes analyzed, *S. yellowstonense* SS-5^T^ contained the highest number of CRISPR arrays at 19, with four to 41 spacer regions in each, while *T. jamiesonii* GBS1^T^ only contained four CRISPR arrays, with four to 15 spacer regions per array. Each of these host genomes had a number of spacer sequences with significant homology to the OS3173, GBS41, and JC39 genomes (Figure 5 and Supplementary Figure S11). These matches were grouped into high confidence matches (i.e., 100% length of the spacer aligned with >90% identity) and lower confidence matches (<100% length of spacer aligned and/or <90% identity). No significant matches of spacer sequences from the putative hosts were detected for Conch37. The six CRISPR spacers from the *T. ruber* OC1/4^T^ genome that had homology to the viral genomes were all somewhat distant and classified as lower confidence matches (80–95% nucleic acid identity, Supplementary Figure S11), which is reasonable considering that this organism was isolated from samples collected from Octopus Spring in 1994 (Huber et al., 1998), and the samples from which the UViGs were assembled were collected in 2007 and 2012. Furthermore, metagenomic studies of the pink streamer community in Octopus Spring revealed three dominant *Thermocrinis* populations, but each was distinct from *T. ruber* OC1/4^T^ (Takacs-Vesbach et al., 2013); thus, it is possible that the *T. ruber* OC1/4^T^ genotype is rarely encountered by members of TOSV. To assess this possibility, we analyzed CRISPR arrays contained within metagenome-assembled genomes (MAGs) of *Thermocrinis* and other *Aquificae*, from Octopus Spring and other springs, as well as arrays in unbinned contigs to identify potential matches to the virus sequences. The MAGs did not contain any CRISPR arrays, likely because contigs including these arrays cannot be binned reliably, presumably due to the non-native nucleotide word frequency associated with the foreign-derived CRISPR spacers (data not shown). However, unbinned contigs from the metagenomes that contained high confidence CRISPR spacer matches showed homology to *Thermocrinis* genomes (Supplementary File S1). Similar to matches to the *T. ruber* OC1/4^T^ isolate genome, CRISPR spacers of the *Hydrogenobaculum* sp. 3684 and *S. yellowstonense* SS-5^T^ genomes showed lower confidence matches to the viral genomes (81–92%) apart from one high confidence match between JC39 and *Hydrogenobaculum* sp. 3684 (Supplementary Figure S11C). By comparison, *T. jamiesonii* GBS1^T^, contained three arrays with five CRISPR spacers in total with high confidence matches and significant identity to the GBS41 genome (Supplementary Figure S11B), providing strong evidence of the virus-host relationship.

The CRISPR spacers mapped to several different genes in the “*Pyrovirus*” genomes; however, the C-terminus of the PolA was targeted by spacers in each virus, albeit mostly low confidence matches, and another two spacers mapped to the central portion of the PolA gene in GBS41, suggesting that the C-terminus of the PolA might be a functionally important antiviral target for the host (Figure 5 and Supplementary Figure S11). Accordingly, the C-terminal-encoding portion of the *polA* gene was among the most highly conserved regions of the genomes (Figure 4). The capsid protein gene of OS3173 and JC39 matched with low confidence to several spacers, but this was not the case for GBS41. The terminase large subunit in JC39 had matches to multiple CRISPR spacers, although this was not observed in the other two UViGs.

*Thermocrinis* is the dominant member of the pink streamer community in Octopus Spring (Reysenbach et al., 1994; Takacs-Vesbach et al., 2013; Colman et al., 2016) and the planktonic community in GBS (Cole et al., 2013); thus, it is reasonable to hypothesize that the natural host for the dominant viruses in these springs is *Thermocrinis*, as supported by shared gene content and CRISPR spacer matches. *Thermocrinis* is also extremely abundant in Little Hot Creek (Vick et al., 2010). Thus, we suggest that TOSV and TGBSV, all encoding the polyprotein (Figure 2), associate with *Thermocrinis* as their putative host. These viruses are typified by OS3173 and GBS41, with the complete UViG of OS3173 serving as the reference genome for the group.

In contrast, *Sulfurihydrogenibium* is the dominant *Aquificae* at Calcite Spring (Reysenbach et al., 2000; Inskeep et al., 2010) and the sample from Joseph’s Coat Spring (JC2E) contains abundant *Hydrogenobaculum* spp. (Inskeep unpl.). We suggest that *Sulfurihydrogenibium* and/or *Hydrogenobaculum* are the most likely hosts for AJCSV and ACSV, especially as multiple homologous matches to spacer regions were obtained to both these potential hosts with the JC39 UViG. *Hydrogenobaculum* forms a distinct clade from *Thermocrinis*, *Hydrogenobacter*, *Aquifex*, and *Hydrogenivirga* within the *Aquificaceae*, and predominates in low pH springs (pH < 4.0) (Inskeep et al., 2013; Takacs-Vesbach et al., 2013). *Sulfurihydrogenibium* belongs to the sister family, *Hydrogenothermaceae*, and predominates in circumneutral springs (pH 6.5–7.8) (Takacs-Vesbach et al., 2013) and grows in a wide pH range in the lab (pH 5.0–8.8) (O’Neill et al., 2008). In this regard, it is noteworthy that some geothermal springs are poorly buffered and can change from circumneutral to highly acidic in both space and time, depending on the amounts and sources of geothermal and meteoric water that pool, and particularly on the source of sulfide, which can be oxidized to sulfuric acid by sulfide- and sulfur-oxidizing microorganisms (Nordstrom et al., 2009). Thus, it is possible that AJCSV and/or ACSV viruses encounter and infect *Sulfurihydrogenibium* in circumneutral regions of the springs and *Hydrogenobaculum* in highly acidic regions, explaining the nearly equal numbers of CRISPR spacer matches in each organism. Additionally, the gene-sharing network and a neighbor-joining tree based on amino acid identity both suggested a distant relationship to *Hydrogenobaculum* phage 1 (Figures 3A,B and Supplementary Figures S8, S9; Gudbergsdóttir et al., 2016), a 19,351 bp UViG recovered from a metagenome from Grensdalur, Iceland that was assigned to *Hydrogenobaculum* based on CRISPR spacer matches to genomes from cultivated *Hydrogenobaculum* strains. Since the exact hosts of these viruses are not conclusive, we suggest the names *Aquificae* Joseph’s Coat Spring Virus (JC39, high-quality draft genome) and *Aquificae* Conch Spring Virus (Conch37, high-quality draft genome) to represent the best genomes of these species.

## Description of Proposed Viruses

(Py.ro.vi’rus. Gr. n. *pur*, fire; N.L. neut. n. Pyrovirus, “fire virus,” a thermophilic virus).

Based on the data presented here, we propose the following names and taxonomic relationships. Multiple genomic features suggest the seven novel UViGs belong to the order *Caudovirales*. The low overall sequence similarity and distinct placement of these taxa in gene-sharing networks suggest these viruses belong to an unclassified viral family and represent one putative genus-level group.

The proposed genus “*Pyrovirus*” accommodates TOSV, TGBSV, AJCSV, and ACSV, with the complete genome of OS3173 serving as the reference species for the genus. Members of this genus are predicted to infect *Aquificae* and are abundant in terrestrial geothermal springs. The estimated size of genomes in this genus range from 37 to 42 kb. The genomes contain genes encoding a thymidylate kinase, a holin, a lytic transglycosylase, a portal protein, large and small subunits of terminase, phage capsid protein, DNA polymerase A (with fused or unfused DUF927 helicase domain), a nuclease and a helicase. Members of this genus are proposed to employ a complex bidirectional replication strategy.

## Data Availability Statement

The datasets presented in this study can be found in online repositories. The names of the repository/repositories and accession number(s) can be found in the article/ Supplementary Material.

## Author Contributions

BH, JD, TS, and DM: conceptualization. MP, BH, SR, PT, and DM: data curation. MP, SR, RD, CS, AM, SM, and TG: formal analysis. MP, SR, PT, JD, ML, SM, and TG: investigation. MP, BH, SR, PT, JD, ML, SM, TG, TS, and DM: methodology. BH, SR, PT, EE-F, and DM: resources. BH and PT: supervision. MP, SR, and PT: validation. MP: visualization. BH, ML, SM, TS, and DM: writing – original draft. MP, BH, SR, PT, RD, CS, AM, JD, ML, SM, TG, TS, EE-F, and DM: writing – review and editing. All authors contributed to the article and approved the submitted version.

## Conflict of Interest

ML and SM were employed by the company Lucigen Corporation. TS was employed by the company Tamarack Bioscience, Inc. DM was employed by the company Varigen Biosciences Corporation. The remaining authors declare that the research was conducted in the absence of any commercial or financial relationships that could be construed as a potential conflict of interest.

## Footnotes

1 http://www.ncbi.nlm.nih.gov/genomes/GenomesGroup.cgi?taxid=10239&host=archaea

2 http://www.ncbi.nlm.nih.gov/genomes/GenomesGroup.cgi?taxid=10239&host=bacteria

3 https://jgi.doe.gov/user-programs/pmo-overview/protocols-sample-preparation-information/jgi-bacterial-dna-isolation-ctab-protocol-2012/

4 https://img.jgi.doe.gov/m/doc/MetagenomeAnnotationSOP.pdf

5 http://blast.ncbi.nlm.nih.gov

6 http://ncbi.nlm.nih.gov/Structure/cdd

7 http://hmmer.org

8 https://toolkit.tuebingen.mpg.de/tools/hhpred

9 https://mafft.cbrc.jp/alignment/server/

10 https://www.ebi.ac.uk/Tools/hmmer/search/hmmsearch

11 http://www.cbs.dtu.dk/services/TMHMM/

12 http://stothard.afns.ualberta.ca/cgview_server/

13 https://bitbucket.org/srouxjgi/inovirus/src/master/

14 https://www.osti.gov/biblio/1241166-bbmap-fast-accurate-splice-aware-aligner

15 http://cytoscape.org

16 https://www.genome.jp/viptree/

17 https://crisprcas.i2bc.paris-saclay.fr/CrisprCasFinder/Index

18 http://enveomics.blogspot.com/2013/01/blasttabcatsbjpl.html

19 https://github.com/lmrodriguezr/enveomics

20 http://img.jgi.doe.gov/m

21 https://www.ncbi.nlm.nih.gov/
