# Supplementary material for: Diversity and Distribution of a Novel Genus of Hyperthermophilic Aquificae Viruses Encoding a Proof-Reading Family-A DNA Polymerase
Source: Front Microbiol. 2020 Nov 12;11:583361. doi: 10.3389/fmicb.2020.583361 (PMC7689252; doi:10.3389/fmicb.2020.583361)
Supplement: Supplementary file 4 [file Data_Sheet_1.PDF]

# *Supplementary Material*

**Supplementary Tables** **Page**


1. Table S1. Minimum Information about additional Uncultivated Virus Genomes 2


**Supplementary Figures**


1. Figure S1. Recruitment of the OS3173 virus genome 3
2. Figure S2. Similarity and NJ phylogeny of 10 most abundant OS contigs 4
3. Figure S3. Phylogenetic placement of 10 most abundant OS contigs 5
4. Figure S4. Recruitment of the GBS41 virus genome 6
5. Figure S5. Similarity and NJ phylogeny of 10 most abundant GBS contigs 7
6. Figure S6. Phylogenetic placement of 10 most abundant GBS contigs 8
7. Figure S7. Sampling sites for metagenomes containing "*Pyrovirus*" contigs 9
8. Figure S8. Phylogenetic placement of 7 UViGs investigated 11
9. Figure S9. Synteny and similarity of Conch37 to *Hydrogenobaculum* phage 1 12
10. Figure S10. ML phylogeny of the terminase large subunit 13
11. Figure S11. CRISPR spacer matches to viral genomes 14


**Supplementary Files**


1. File S1. Classification and CRISPR results
2. File S2. Protein annotation and homology search results
3. File S3. Composite annotations across near-complete UViGs

## Table S1. Minimum Information about additional Uncultivated Virus Genomes (MIUViG).

| Metadata | Oct28 | Conch32 | Calcite32 |
|---|---|---|---|
| **Source of UViG** | Metagenome (not viral targeted) | Metagenome (not viral targeted) | Metagenome (not viral targeted) |
| **Sequencing approach** | Illumina HiSeq 2000, Illumina HiSeq 2500 | Illumina HiSeq 2000 | Sanger |
| **Assembly software** | SPAdes v 3.10.0 (--meta --only-assembler -k 21, 33, 55, 77, 99, 127) | SPAdes v 3.10.0 (--meta --only-assembler -k 21, 33, 55, 77, 99, 127) | Phrap |
| **Viral identification software** | Viral polA BLAST | Viral polA BLAST | Viral polA BLAST |
| **Predicted genome type** | dsDNA | dsDNA | dsDNA |
| **Predicted genome structure** | Non-segmented | Non-segmented | Non-segmented |
| **Detection type** | Independent sequence (UViG) | Independent sequence (UViG) | Independent sequence (UViG) |
| **Assembly quality** | Genome fragment | Genome fragment | Genome fragment |
| **Number of contigs** | 1 | 1 | 1 |

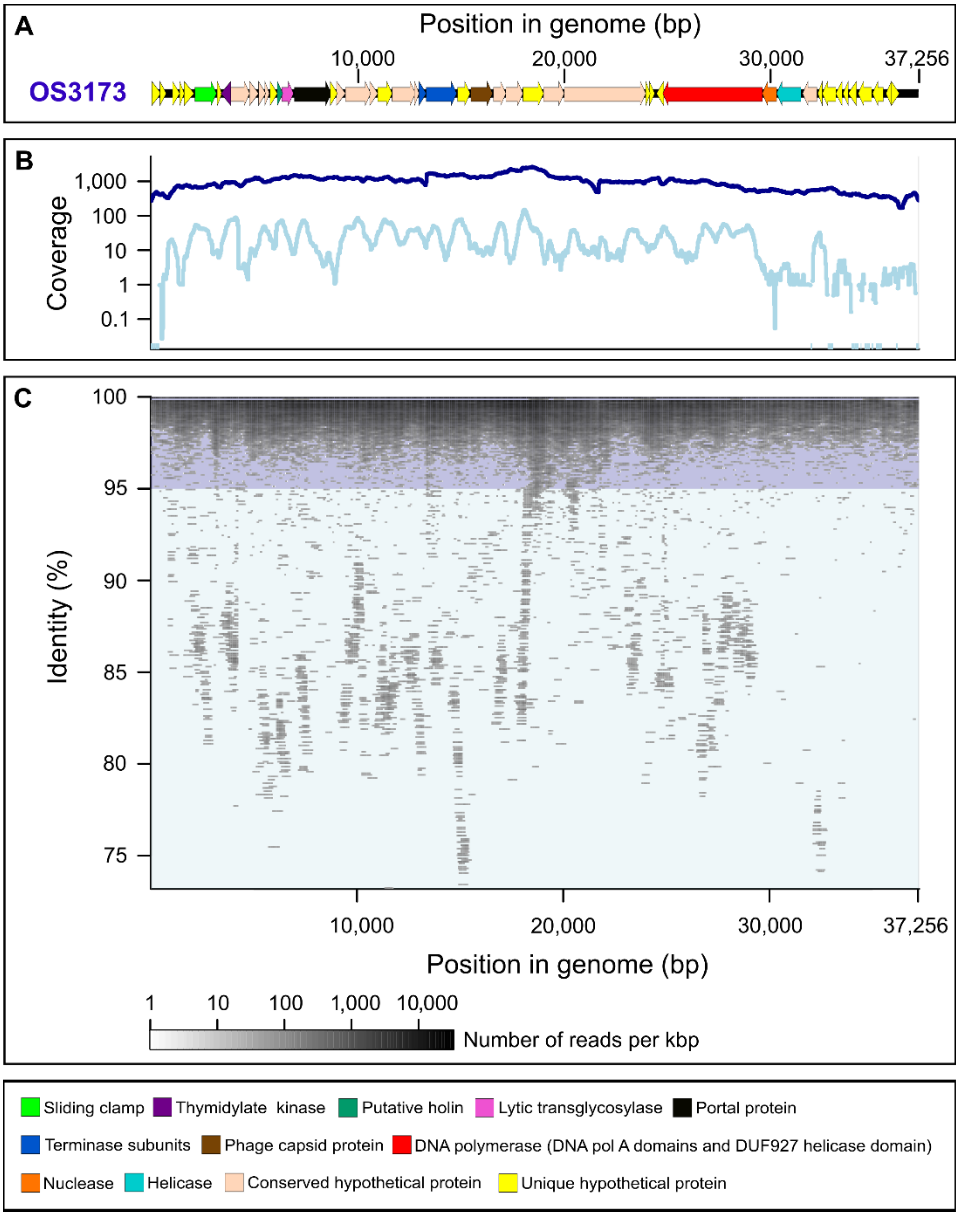


**Figure S1. Recruitment of the OS3173 virus genome.** (A) Linearized map of the OS3173 genome. Arrows denote putative direction of transcription. Genes are color coded as shown in the bottom panel. (B) Coverage across the genome at 95% nucleotide identity (dark blue) and between 95% and 80% nucleotide identity (light blue). (C) Plot of individual reads across the genome. Shades of blue correspond to coverage shown in panel B.

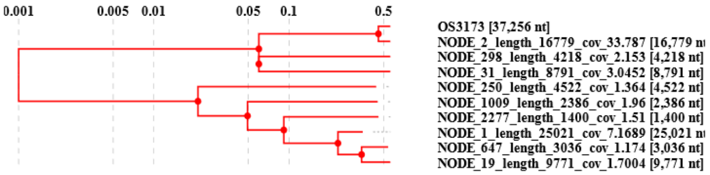


Normalized tBLASTx Score

| | OS3173 | NODE_2 | NODE_298 | NODE_31 | NODE_250 | NODE_1009 | NODE_2277 | NODE_1 | NODE_647 | NODE_19 |
|---|---|---|---|---|---|---|---|---|---|---|
| OS3173 | 1 | 0.7951 | 0 | 0 | 0 | 0 | 0 | 0 | 0 | 0 |
| NODE_2 | 0.7951 | 1 | 0 | 0 | 0 | 0 | 0 | 0 | 0 | 0 |
| NODE_298 | 0 | 0 | 1 | 0 | 0 | 0 | 0 | 0 | 0 | 0 |
| NODE_31 | 0 | 0 | 0 | 1 | 0 | 0 | 0 | 0 | 0 | 0 |
| NODE_250 | 0 | 0 | 0 | 0 | 1 | 0.1152 | 0.1059 | 0.3545 | 0 | 0 |
| NODE_1009 | 0 | 0 | 0 | 0 | 0.1152 | 1 | 0.173 | 0.3647 | 0 | 0 |
| NODE_2277 | 0 | 0 | 0 | 0 | 0.1059 | 0.173 | 1 | 0.4721 | 0 | 0 |
| NODE_1 | 0 | 0 | 0 | 0 | 0.3545 | 0.3647 | 0.4721 | 1 | 0.6279 | 0.4847 |
| NODE_647 | 0 | 0 | 0 | 0 | 0 | 0 | 0 | 0.6279 | 1 | 0.5878 |
| NODE_19 | 0 | 0 | 0 | 0 | 0 | 0 | 0 | 0.4847 | 0.5878 | 1 |

**Figure S2.** Similarity matrix inferred from normalized tBLASTx scores and associated neighbor-joining tree of ten viral contigs with the highest coverage from the Octopus Spring virus-enriched metagenome. Contig length and sequence identifiers are noted on the labels on the distance tree. A phylogenetic analysis relating these contigs to known dsDNA viral genomes is shown in Figure S3. Overall, the ten contigs with the highest coverage obtained from the Octopus Spring virus-enriched metagenome were grouped into at least four distinct clusters based on the viral proteomic tree approach. The first cluster contains OS3173 (TOSV) together with contig NODE 2 (also visible in Figure 4, members of "*Pyrovirus*"), two singleton clusters consisting of NODE 298 and NODE 31, respectively, and a large cluster containing NODE 1 (see Figure 4, S3), NODE 250, NODE 1009, NODE 2277, NODE 647 and NODE 19 related to *Pyrobaculum* spherical virus and *Thermoproteus tenax* spherical virus 1 (Figure 4).

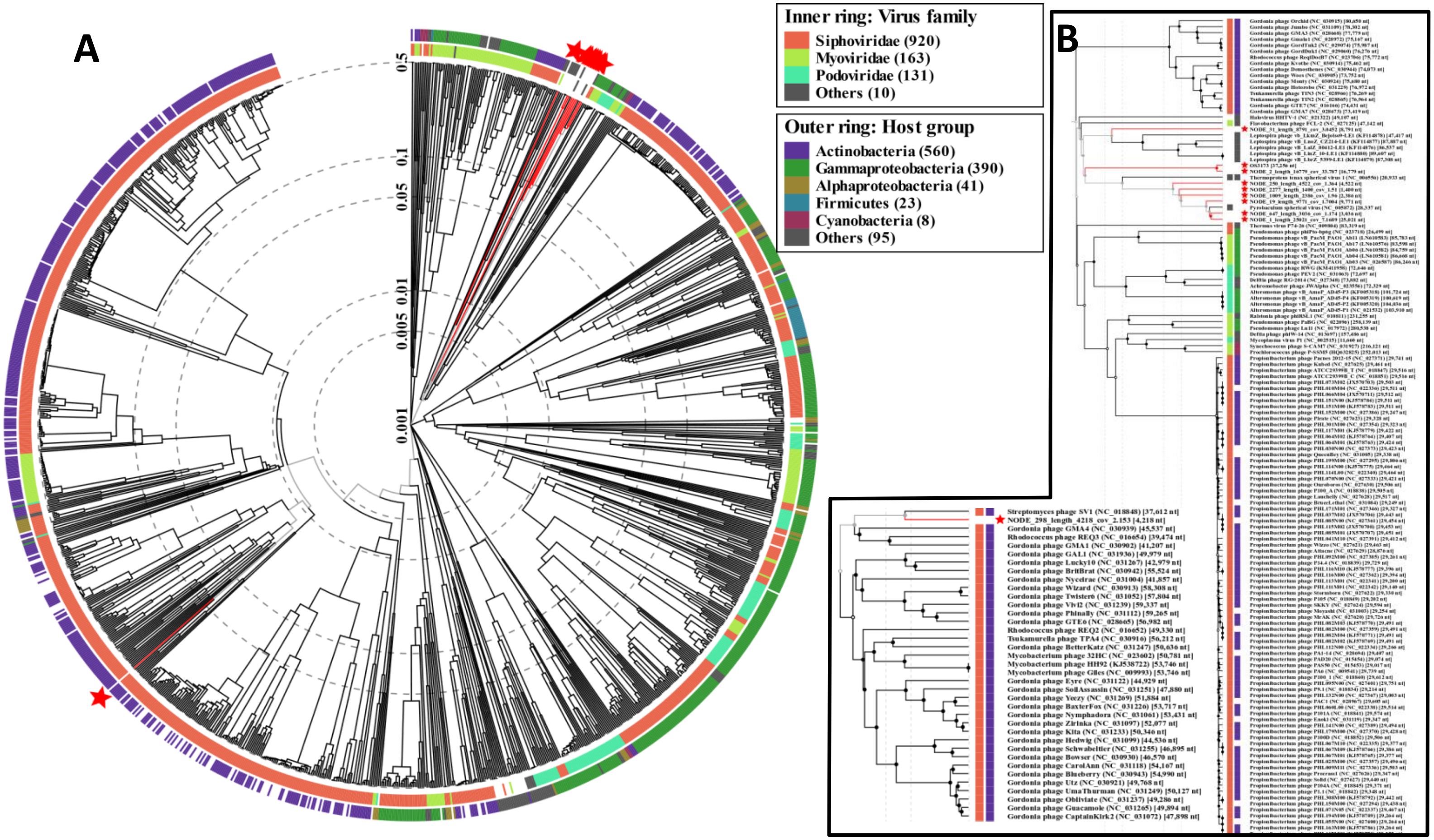


**Figure S3.** (A) Neighbor-joining tree of ten viral contigs with the highest coverage from Octopus Spring virus-enriched metagenome within the context of other dsDNA viral genomes. The placement of the ten contigs with the highest coverage from the Octopus Spring virus-enriched metagenome is indicated with red stars. (B) Subtrees containing these 10 viral contigs, indicated in red, with their closest relatives. The same clusters were obtained as from results of the gene-sharing network together with the tBLASTx relationships. The first cluster contains OS3173(TOSV) together with contig NODE 2 (also visible in Figure 4, members of "*Pyrovirus*"), and groups as sister to the large cluster containing NODE 1 (see Figure 4), NODE 250, NODE 1009, NODE 2277, NODE 647, NODE 19, *Pyrobaculum* spherical virus and *Thermoproteus tenax* spherical virus 1 (Figure 4). The two singletons were grouped with *Leptospira* and *Streptomyces* phages, respectively.

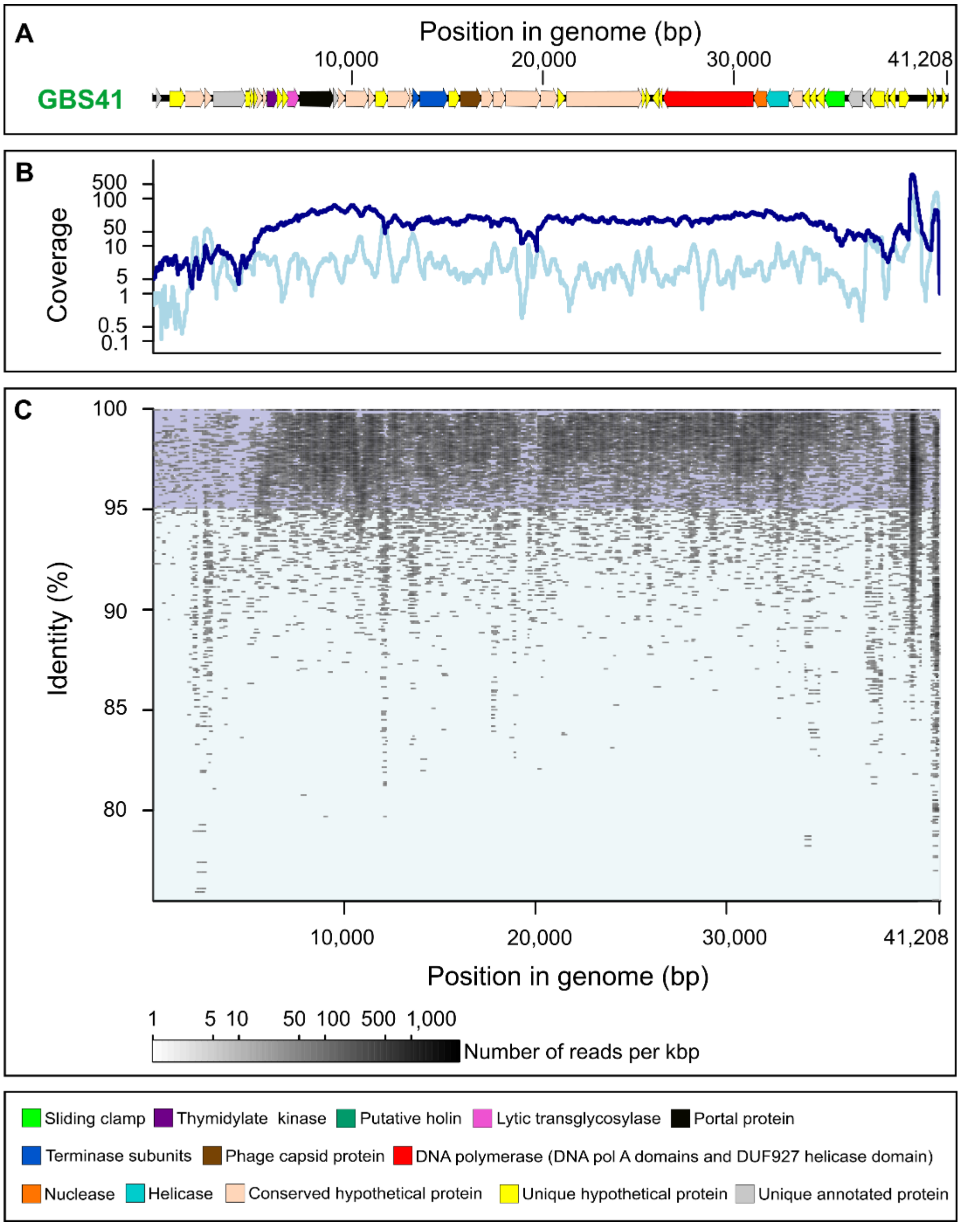


**Figure S4. Recruitment of the GBS41 virus genome.** (A) Linearized map of the GBS41 genome. Arrows denote putative direction of transcription. Genes are color coded as shown in the bottom panel. (B) Coverage across the genome at 95% nucleotide identity (dark blue) and between 95% and 80% nucleotide identity (light blue). (C) Plot of individual reads across the genome. Shades of blue correspond to coverage shown in panel B.

10 Most abundant viruses GBS - Related viruses

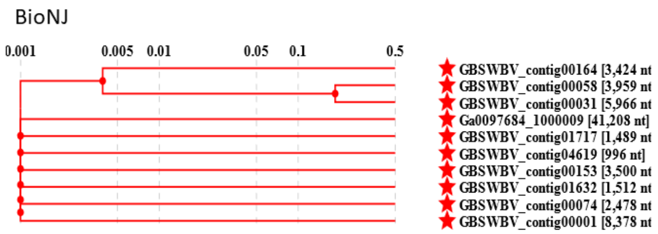


Normalized tBLASTx Score

| | contig00164 | contig00058 | contig00031 | Ga0097684 | contig01717 | contig04619 | contig00153 | contig01632 | contig00074 | contig00001 |
|---|---|---|---|---|---|---|---|---|---|---|
| GBSWBV_contig00164 | 1 | 0.0178 | 0 | 0 | 0 | 0 | 0 | 0 | 0 | 0 |
| GBSWBV_contig00058 | 0.0178 | 1 | 0.3706 | 0 | 0 | 0 | 0 | 0 | 0 | 0 |
| GBSWBV_contig00031 | 0 | 0.3706 | 1 | 0 | 0 | 0 | 0 | 0 | 0 | 0 |
| Ga0097684_1000009 | 0 | 0 | 0 | 1 | 0 | 0 | 0 | 0 | 0 | 0 |
| GBSWBV_contig01717 | 0 | 0 | 0 | 0 | 1 | 0 | 0 | 0 | 0 | 0 |
| GBSWBV_contig04619 | 0 | 0 | 0 | 0 | 0 | 1 | 0 | 0 | 0 | 0 |
| GBSWBV_contig00153 | 0 | 0 | 0 | 0 | 0 | 0 | 1 | 0 | 0 | 0 |
| GBSWBV_contig01632 | 0 | 0 | 0 | 0 | 0 | 0 | 0 | 1 | 0 | 0 |
| GBSWBV_contig00074 | 0 | 0 | 0 | 0 | 0 | 0 | 0 | 0 | 1 | 0 |
| GBSWBV_contig00001 | 0 | 0 | 0 | 0 | 0 | 0 | 0 | 0 | 0 | 1 |

**Figure S5.** Distance matrix and neighbor-joining tree of ten viral contigs with the highest coverage from the Great Boiling Spring viral metagenome. Contig length and sequence identifiers are noted on the labels on the distance tree with sequence identifiers deposited in the DOE-JGI IMG/M. A Neighbor-Joining phylogenetic analysis relating these contigs to known dsDNA viral genomes is shown in Figure S6. The ten contigs with the highest coverage obtained from the Great Boiling Spring virus-enriched metagenome were grouped into eight distinct clusters based on the viral proteomic tree approach. The first cluster contains contig00164, contig00058 and contig00031, with GBS41 (TGBSV, Ga0097684_1000009) as the sole member of "*Pyrovirus*", while all other contigs showed no similarity among them based on normalized tBLASTx scores.

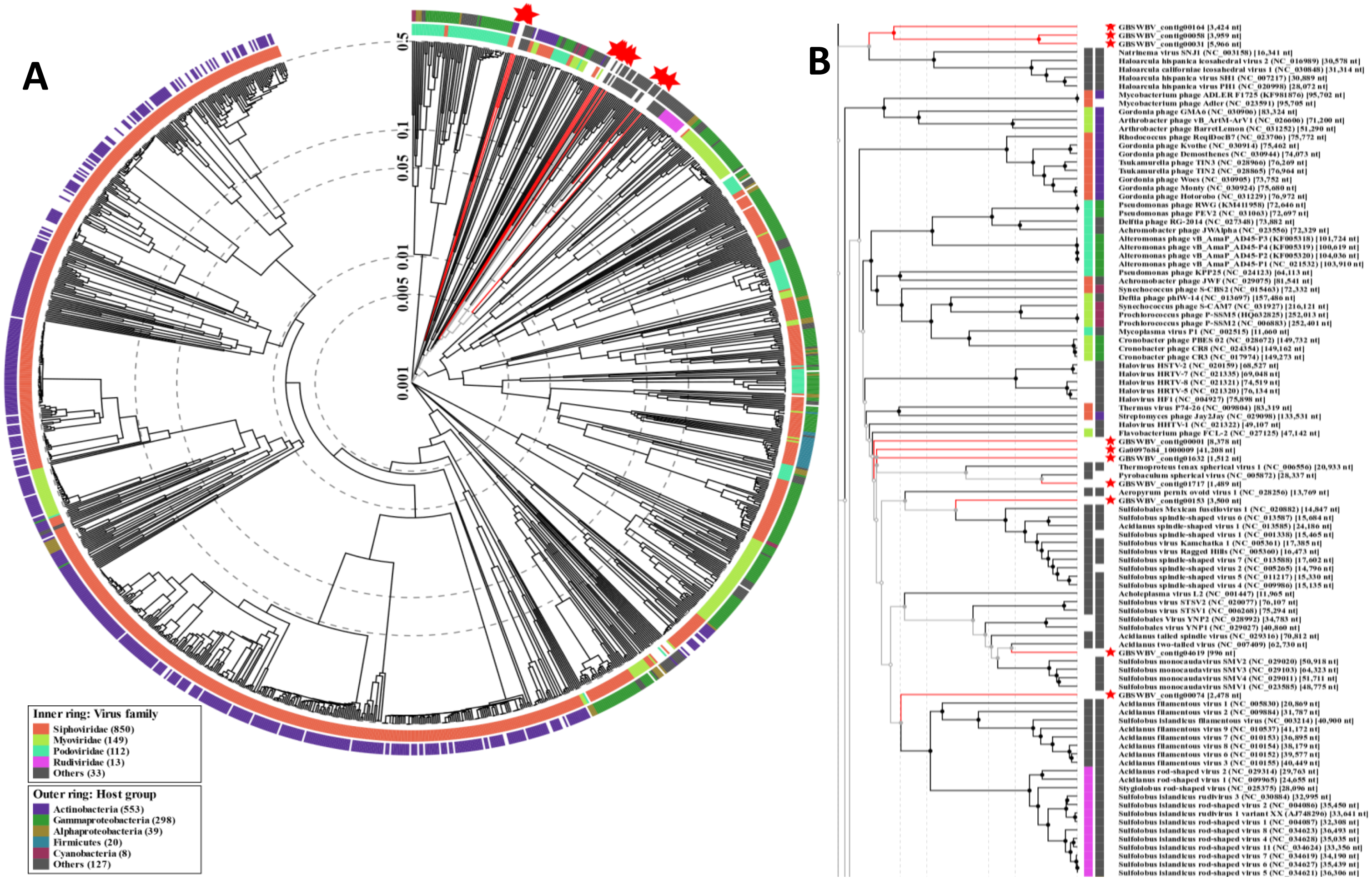


**Figure S6.** (A) Neighbor-joining tree of the ten viral contigs with the highest coverage from the Great Boiling Spring (GBS) viral metagenome within the context of other dsDNA viral genomes. (B) Subtree containing these 10 GBS viral contigs indicated in red, with their closest relatives. A single grouping of contig00164, contig00058 and contig00031 was placed on a distinct branch as sister to *Natrinema* and *Haloarcula* viruses, contig01717 were placed as sister to *Pyrobaculum* spherical virus, three contigs were placed as somewhat related to *Sulfolobales* and *Sulfolobus* viruses, while the placement of the remaining contigs were uncertain. However, the placement of all of these viral contigs, with the exception of GBS41, are not reliable due to the small size of these contigs.

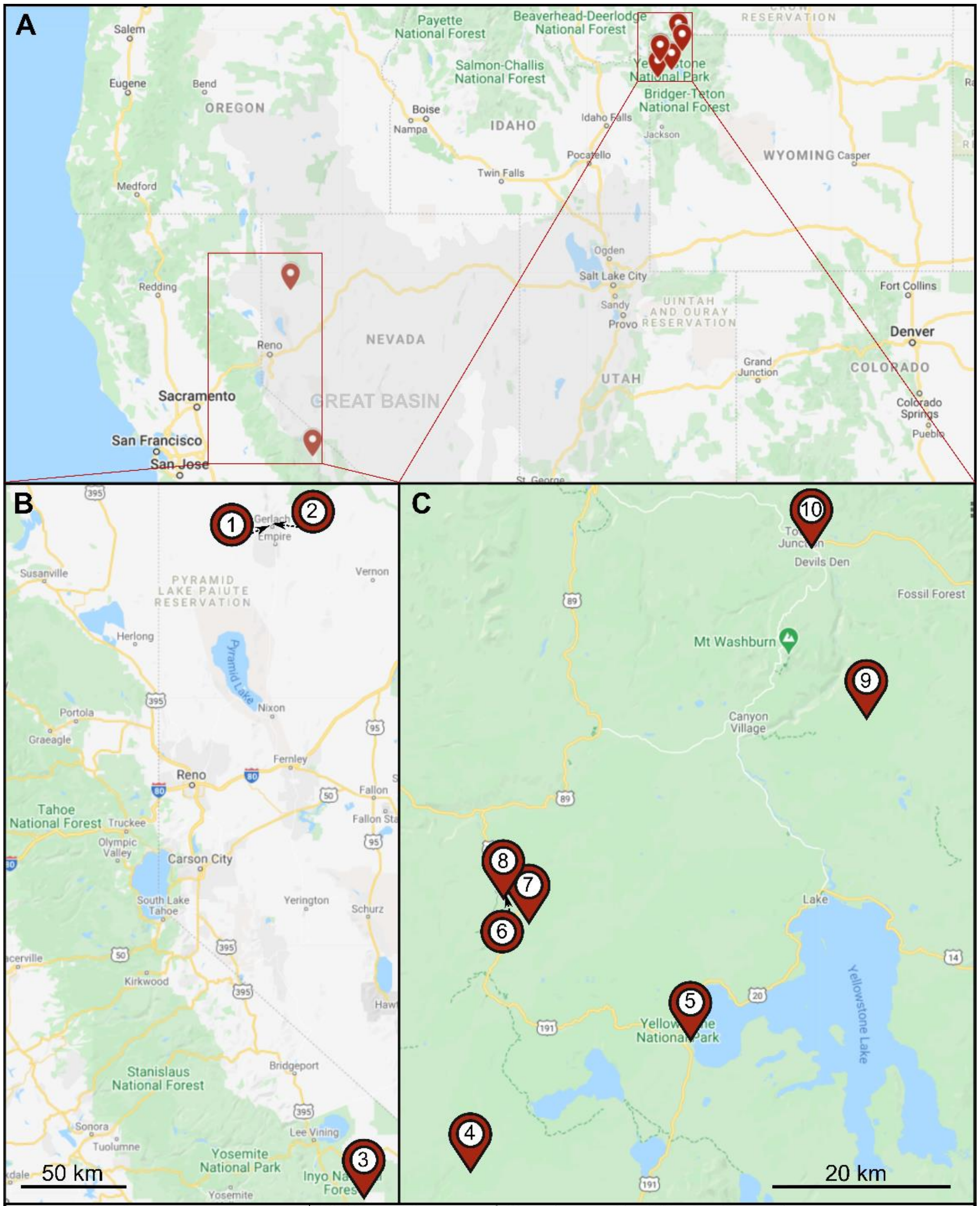


| | Spring | DNA pol A group | Dominant *Aquificales* in spring |
|---|---|---|---|
| Great Basin | ① Sandy's Spring West | TGBSV | *Thermocrinis** |
| | ② Great Boiling Spring | TGBSV | ***Thermocrinis*** |
| | ③ Little Hot Creek | TGBSV | ***Thermocrinis*** |
| Yellowstone National Park | ④ Bechler Spring | TOSV | ***Thermocrinis*** |
| | ⑤ Black Pool | TOSV | *Thermocrinis* + |
| | ⑥ Conch Spring | TOSV, ACSV | *Thermocrinis* * |
| | ⑦ Octopus Spring | TOSV | ***Thermocrinis*** |
| | ⑧ Bath Spring | TOSV | *Thermocrinis* * |
| | ⑨ Joseph's Coat Spring | AJCSV | *Hydrogenobaculum* * |
| | ⑩ Calcite Spring | AJCSV | ***Sulfurihydrogenibium*** |

+ Based on literature
* At least 500 genes assigned to genus with >90% identity (distribution by BLAST percentage identities)
**Dominant *Aquificales* based on literature and distribution by BLAST percentage identities**

**Figure S7.** (A) Overview of the area where "*Pyrovirus*" contigs were identified from. (B) Sampling sites for metagenomes from the U.S. Great Basin from which "*Pyrovirus*" contigs were identified. All three contigs from this area belonged to the proposed species *Thermocrinis* Great Boiling Spring Virus (TGBSV) based on the DNA PolA phylogeny. *Thermocrinis* is the dominant member of *Aquificales* in the spring community in all three springs. (C) Sampling sites for metagenomes from Yellowstone National Park from which "*Pyrovirus*" contigs were identified. Contigs putatively assigned as belonging to the proposed species *Thermocrinis* Octopus Spring Virus (TOSV) based on the DNA PolA phylogeny were identified from Bechler Spring, Black Pool, Conch Spring, Octopus Spring and Bath Spring. *Thermocrinis* represent the dominant *Aquificales* within the microbial communities all five these springs. Contigs putatively assigned to *Aquificae* Joseph's Coat Spring Virus (AJCSV) were identified from Joseph's Coat Spring and Calcite Spring, and *Sulfurihydrogenibium* and *Hydrogenobaculum* may represent dominant *Aquificales* within these communities. The sole contig assigned to the proposed species *Aquificae* Conch Spring Virus (ACSV) were also identified from Conch Spring. Dominant *Aquificales* were determined based on literature ($^{+}$), distribution by BLAST percentage identities as incorporated in IMG (*), or by both these approaches where data were available (boldface).

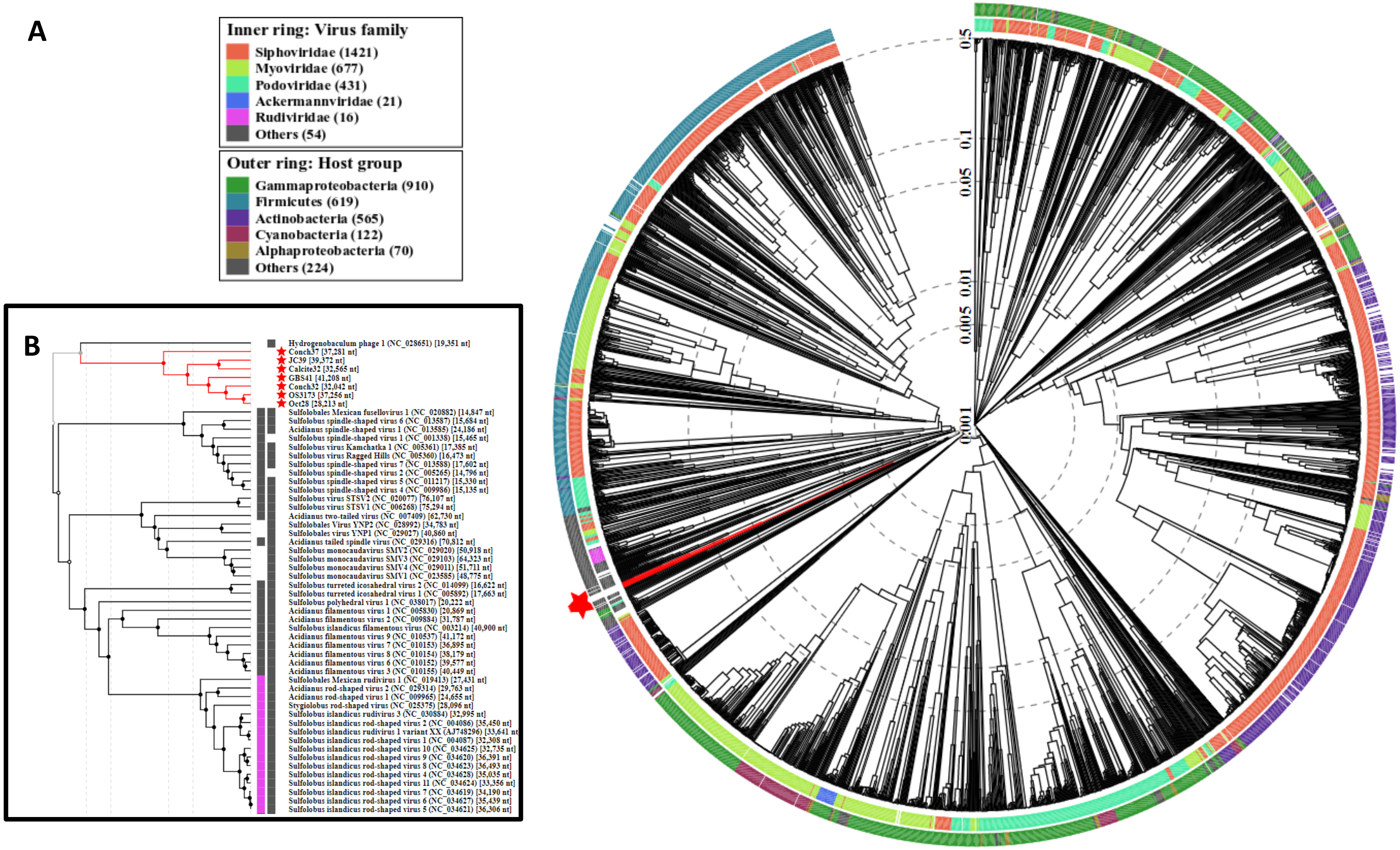


**Figure S8.** Relationships between UViGs are inferred from normalized tBLASTx scores using the viral proteomic tree approach. (A) Neighbor-joining tree of the OS3173-like UViGs within the context of other dsDNA viral genomes. The red star denotes the placement of members of the putative novel genus "*Pyrovirus*". (B) Subtree containing the OS3173-like UViGs and closest related dsDNA viral genomes. Of all available dsDNA viral reference sequences, only *Hydrogenobaculum* phage HP1 showed any similarity to members of the putative genus "*Pyrovirus*", with a very low normalized tBLASTx score of 0.02 to Conch37 (see Figure S9).

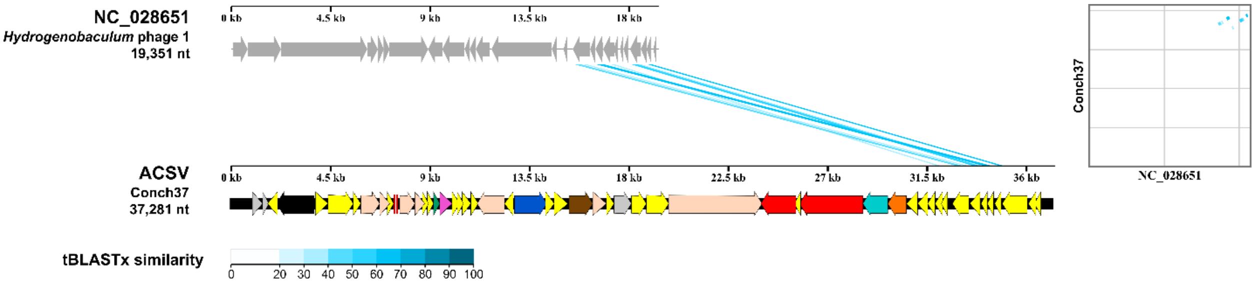


**Figure S9.** Synteny and amino acid identity between Conch37/ACSV and *Hydrogenobaculum* phage 1 based on tBLASTx score determined with ViPtree.

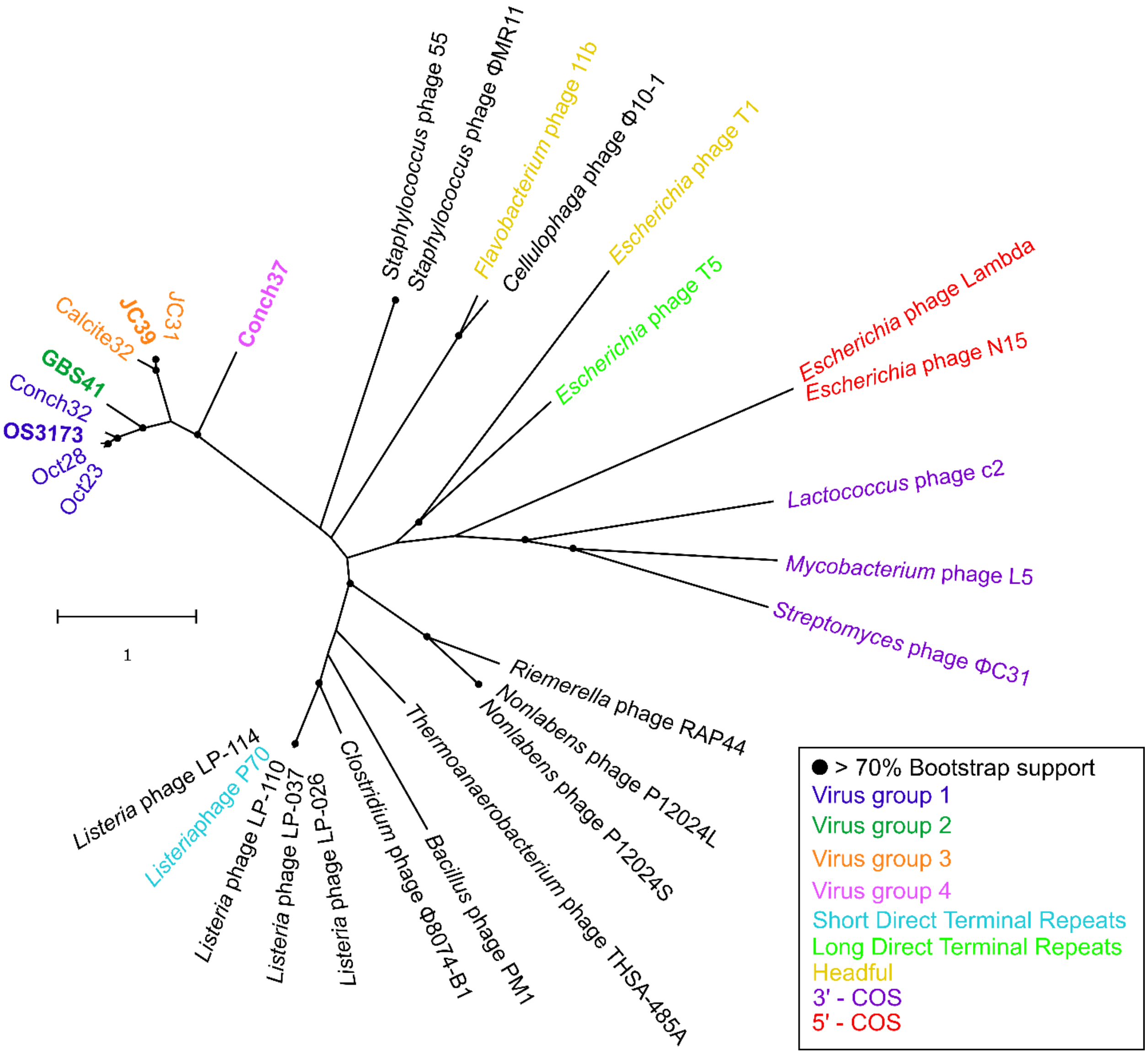


**Figure S10.** Maximum-likelihood tree of the protein sequences of the large subunits for the terminase. Branch support was inferred from 1,000 bootstrap pseudoreplicates. Reference sequences from Chelikani et al., 2014 was used.

**A**

**OS3173**

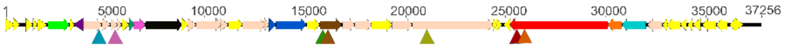


*Thermocrinis ruber*

| CRISPR cluster | Spacer | Length (nt) | Position in *T. ruber* | % Identity | Position in OS3173 | % Length |
|---|---|---|---|---|---|---|
| | 4-4 | 36 | 806,209 - 806,244 | 81% | 15,593 - 15,628 | 100% |
| | 6-1 | 44 | 906,243 - 906,286 | 95% | 25,139 - 25,182 | 84% |
| | 6-2 | 44 | 906,310 - 906,353 | 94% | 4,754 - 4,797 | 84% |
| | 6-3 | 43 | 906,377 - 906,419 | 94% | 15,785 - 15,829 | 81% |
| | 6-8 | 43 | 906,710 - 906,752 | 94% | 25,667 - 25,710 | 84% |

*Thermocrinis jamiesonii*

| CRISPR cluster | Spacer | Length (nt) | Position in *T. jamiesonii* | % Identity | Position in OS3173 | % Length |
|---|---|---|---|---|---|---|
| | 1-2-3 | 37 | Scaffold 1: 357,400 - 357,437 | 80% | 21,435 - 21,464 | 81% |

*Hydrogenobaculum* sp. 3684

| CRISPR cluster | Spacer | Length (nt) | Position in *Hydrogenobaculum* | % Identity | Position in OS3173 | % Length |
|---|---|---|---|---|---|---|
| | 8-12 | 38 | 1,089,516 - 1,089,554 | 83% | 5,516 - 5,551 | 95% |

**B**

**GBS41**

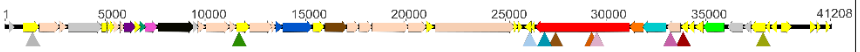


*Thermocrinis jamiesonii*

| CRISPR cluster | Spacer | Length (nt) | Position in *T. jamiesonii* | % Identity | Position in GBS41 | % Length |
|---|---|---|---|---|---|---|
| | 1-2-1 | 39 | Scaffold 1: 357,264 - 357,302 | 90% | 11,991 - 12,029 | 100% |
| | 1-2-2 | 39 | Scaffold 1: 357,332 - 357,370 | 100% | 26,913 - 26,951 | 100% |
| | 1-2-4 | 39 | Scaffold 1: 357,466 - 357,504 | 87% | 33,896 - 33,934 | 100% |
| | 2-1-10 | 40 | Scaffold 2: 676 - 715 | 100% | 27,311 - 27,350 | 100% |
| | 3-1-1 | 37 | Scaffold 3: 4,303 - 4,340 | 82% | 29,351 - 29,384 | 92% |
| | 3-1-7 | 43 | Scaffold 3: 4,705 - 4,747 | 86% | 37,853 - 37,896 | 100% |
| | 3-2-2 | 35 | Scaffold 3: 50,167 - 50,201 | 100% | 33,205 - 33,239 | 100% |
| | 3-2-4 | 40 | Scaffold 3: 50,297 - 50,336 | 97% | 29,408 - 29,447 | 100% |

*Thermocrinis ruber*

| CRISPR cluster | Spacer | Length (nt) | Position in *T. ruber* | % Identity | Position in GBS41 | % Length |
|---|---|---|---|---|---|---|
| | 4-5 | 37 | 357,332 - 357,369 | 86% | 25,499 - 25,535 | 100% |

*Sulfurihydrogenibium yellowstonense*

| CRISPR cluster | Spacer | Length (nt) | Position in *S. yellowstonense* | % Identity | Position in GBS41 | % Length |
|---|---|---|---|---|---|---|
| | 186-1-13 | 36 | Scaffold 186: 1,273 - 1,309 | 80% | 1,209 - 1,238 | 83% |

**C**

**JC39**

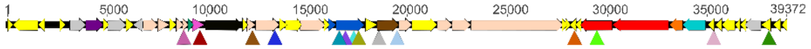


*Hydrogenobaculum* sp. 3684

| CRISPR cluster | Spacer | Length (nt) | Position in *Hydrogenobaculum* | % Identity | Position in JC39 | % Length |
|---|---|---|---|---|---|---|
| | 4-4 | 36 | 591,648 - 591,683 | 83% | 38,536 - 38,507 | 83% |
| | 4-9 | 36 | 591,977 - 592,013 | 89% | 17,684 - 17,719 | 100% |
| | 5-12 | 37 | 928,713 - 928,749 | 87% | 9,664 - 9,693 | 81% |
| | 6-6 | 37 | 931,064 - 931,110 | 81% | 12,184 - 12,220 | 100% |
| | 7-1 | 36 | 1,023,918 - 1,023,953 | 85% | 28,194 - 28,227 | 94% |
| | 7-6 | 37 | 1,024,250 - 1,024,286 | 91% | 17,759 - 17,795 | 100% |
| | 8-12 | 38 | 1,089,516 - 1,089,553 | 81% | 8,879 - 8,914 | 95% |

*Sulfurihydrogenibium yellowstonense*

| CRISPR cluster | Spacer | Length (nt) | Position in *S. yellowstonense* | % Identity | Position in JC39 | % Length |
|---|---|---|---|---|---|---|
| | 17-2-2 | 36 | Scaffold 17: 12,312 - 12,348 | 88% | 35,218 - 35,251 | 94% |
| | 21-1-2 | 37 | Scaffold 21: 2,056 - 2,093 | 81% | 19,644 - 19,680 | 100% |
| | 64-1-6 | 36 | Scaffold 64: 10,495 - 10,531 | 81% | 18,346 - 18,377 | 89% |
| | 94-1-2 | 36 | Scaffold 94: 121 - 157 | 83% | 29,385 - 29,414 | 83% |
| | 94-1-10 | 37 | Scaffold 94: 646 - 682 | 86% | 17,064 - 17,099 | 97% |
| | 121-1-16 | 37 | Scaffold 121: 10,168 - 10,205 | 83% | 13,611- 13,641 | 84% |
| | 209-1-11 | 37 | Scaffold 209: 3,525 - 3,562 | 86% | 17,643 - 17,679 | 100% |

Genes: Sliding clamp; Thymidylate kinase; Putative holin; Lytic transglycosylase; Portal protein; Terminase subunits; Phage capsid protein; DNA polymerase (DNA pol A domains and DUF927 helicase domain); Nuclease; Helicase; Conserved hypothetical protein; Unique hypothetical protein; Unique annotated protein

**Figure S11.** CRISPR spacer matches between viruses and *Aquificae* genomes. (A) Linearized map of the OS3173 genome with sites matching *Thermocrinis ruber* OC1/4$^T$, *Thermocrinis jamiesonii* GBS1$^T$, and *Hydrogenobaculum* sp. 3684 CRISPR spacer sequences denoted by triangles, and schematic and data on matching spacers. (B) Similar plot of the GBS41 genome with sites matching *Thermocrinis jamiesonii* GBS1$^T$, *Thermocrinis ruber* OC1/4$^T$, and *Sulfurihydorgenibium yellowstonense* SS-5$^T$ CRISPR spacers. (C) Linearized map of the JC39 genome with corresponding CRISPR spacer sequence matches to *Hydrogenobaculum* sp. 3684 and *Sulfurihydorgenibium yellowstonense* SS-5$^T$. OS3173, GBS41, and Conch37 represent TOSV, TGBSV, and ACSV, respectively.
